# Co-ordinated spatial propagation of blood plasma clotting and fibrinolytic fronts

**DOI:** 10.1371/journal.pone.0180668

**Published:** 2017-07-07

**Authors:** Ansar S. Zhalyalov, Mikhail A. Panteleev, Marina A. Gracheva, Fazoil I. Ataullakhanov, Alexey M. Shibeko

**Affiliations:** 1 Center for Theoretical Problems of Physicochemical Pharmacology RAS, Moscow, Russia; 2 National Scientific and Practical Centre of Pediatric Hematology, Oncology and Immunology, Moscow, Russia; 3 Department of Physics, Moscow State University, Moscow, Russia; 4 Faculty of Biological and Medical Physics, Moscow Institute of Physics and Technology, Dolgoprudny, Russia; University of Pennsylvania Perelman School of Medicine, UNITED STATES

## Abstract

Fibrinolysis is a cascade of proteolytic reactions occurring in blood and soft tissues, which functions to disintegrate fibrin clots when they are no more needed. In order to elucidate its regulation in space and time, fibrinolysis was investigated using an in vitro reaction-diffusion experimental model of blood clot formation and dissolution. Clotting was activated by a surface with immobilized tissue factor in a thin layer of recalcified blood plasma supplemented with tissue plasminogen activator (TPA), urokinase plasminogen activator or streptokinase. Formation and dissolution of fibrin clot was monitored by videomicroscopy. Computer systems biology model of clot formation and lysis was developed for data analysis and experimental planning. Fibrin clot front propagated in space from tissue factor, followed by a front of clot dissolution propagating from the same source. Velocity of lysis front propagation linearly depended on the velocity clotting front propagation (correlation r^2^ = 0.91). Computer model revealed that fibrin formation was indeed the rate-limiting step in the fibrinolysis front propagation. The phenomenon of two fronts which switched the state of blood plasma from liquid to solid and then back to liquid did not depend on the fibrinolysis activator. Interestingly, TPA at high concentrations began to increase lysis onset time and to decrease lysis propagation velocity, presumably due to plasminogen depletion. Spatially non-uniform lysis occurred simultaneously with clot formation and detached the clot from the procoagulant surface. These patterns of spatial fibrinolysis provide insights into its regulation and might explain clinical phenomena associated with thrombolytic therapy.

## Introduction

Blood coagulation and fibrinolysis are two interconnected networks of proteolytic reactions that control formation and dissolution of fibrin clots, respectively[1]. Both these networks are organized as cascades with numerous positive and negative feedback loops[2,3]. Coagulation is triggered by tissue factor (TF), a transmembrane glycoprotein expressed at the sites of vascular injury. Binding of circulating serine protease factor VIIa to TF turns it into a functional enzyme, which starts coagulation cascade ultimately leading to the formation of thrombin that converts protein fibrinogen into fibrin that polymerizes to form blood clot.

When the clot is no longer needed, it is proteolytically degraded by another protease plasmin produced by the action of fibrinolytic network. This smaller cascade can be initiated by either tissue plasminogen activator (TPA) released by the vascular wall or urokinase plasminogen activator (UPA) present in a precursor form in blood [4]. A critical trigger and cofactor for their action is fibrin itself that not only protects plasmin from inactivation [5], but also accelerates action of TPA by an order of 500 [6].

An essential property shared by coagulation and fibrinolysis is that functioning of these systems is spatially heterogeneous: enzymes formed in one place are transported by diffusion and flow of the fluid to another location[7]. Moreover, the task itself is spatial: clots should be formed strictly at the site of injury, and their dissolution also has to be controlled[8–10]. This appears to be a crucial point for their regulation: there are many examples that roles of individual reactions change in space and time[11,12], or in the presence of flow[13,14]. There are some reports on the critical importance of diffusion-controlled process in fibrinolysis[15], but this system is understood not as good as clotting. There are no reports on sensitivity analysis for fibrinolysis (even without transport process) similar to the ones that are abundant in clotting[3,16,17], and, in general, the meaning of individual components, feedbacks, etc. in fibrinolysis in respect to its spatial distribution still remains not completely clarified.

The objective of the present study was to fill this gap by investigating spatial fibrinolysis. We used a videomicroscopic reaction-diffusion in vitro experimental model to monitor blood clotting initiated by immobilized TF [18–21] in blood plasma supplemented with plasminogen activator. A computational systems biology model of clot formation and dissolution was developed for analysis and interpretation of experimental data. The obtained results suggest a new concept of fibrinolysis regulation, where the critical rate-limiting element is formation of fibrin itself.

## Materials and methods

### Materials

Streptokinase (SK) from Streptococcus (PN S3134) was purchased from Sigma (Saint Louis, MO). TPA (Actilyse) was from Boehringer (Ingelheim, Germany). UPA was from American Diagnostica (Stamford, CT). Thrombodynamics assay kit (HEPES, corn trypsin inhibitor (CTI), Ca(CH_3_COO)_2,_ TF-bearing surface) was from Hemacore (Moscow, Russia). Human recombinant TF (Recombiplastin 2G, Instrumentation Laboratory Company, Bedford, MA, USA) was immobilized by Hemacore LLC using the method that was described in[22] with modifications. Heparin (unfractionated heparin) was from Ferane (Moscow, Russia). Activated factor XI was from Haematologic Technologies (Essex Junction, Vermont). Phospholipids (Phospholipid-TGT) were from Rossix (Mölndal, Sweden).

### Whole blood and plasma preparation

Plasma was prepared from the whole blood of healthy volunteers and patients and collected with the approval of the ethics committees of the Center for Theoretical Problems of Physicochemical Pharmacology and the National Scientific and Practical Centre of Pediatric Hematology, Oncology and Immunology. Blood was drawn into 3.8% sodium citrate (pH 5.5) at a 9:1 blood/anticoagulant volume ratio. Blood was centrifuged at 100 × g for 5 min and the supernatant was collected to obtain platelet-rich plasma (PRP). Platelet count was measured and then PRP was diluted with platelet free plasma (PFP) to set platelet count to 300,000 platelets/μl.

Blood was centrifuged at 1600 × g for 15 min, and the supernatant was collected to obtain platelet-poor plasma (PPP). Supernatant was centrifuged again at 10,000 × g for 5 min to obtain PFP. PPP and PFP were pooled and frozen at -80°C. Pools of plasma from 5 donors each were prepared and used in the experiments.

### Cell culture

A human fetal lung fibroblast line was obtained from the Ivanovskii Research Institute of Virology (Moscow, Russia). The polyethylene terephthalate films with fibroblast monolayer were prepared as described previously[18,20]. The density for cell monolayers was approximately 1000 cells/mm^2^, and its variation (SD) did not exceed 10% among either the different films for each series or different portions of each film. The average density of functional TF on the films was measured using the Actichrome-TF kit (Sekisui Diagnostics, Lexington, MA, USA) and it was 140±15 pmole/m^2^.

### Patient

BE, male, 1.5 yo, with juvenile myelomonocytic leukemia exhibited an occlusive catheter-associated thrombosis of the left iliac vein, was treated with TPA 0.03 mg/kg/h and heparin 10 IU/kg/h. Detailed description of the patient can be found in S1 Appendix.

### Spatial clot growth and lysis experiments

Clot growth and lysis experiments were performed using the video-microscopy device and image collecting software from the Thombodynamics assay as described in[21] with modifications. Frozen plasma was thawed at 37°C within 30 minutes; then it was supplemented with HEPES at 30 mmol/L to stabilize pH at 7.4 during the experiment and CTI at 0.2 mg/ml final concentration to inhibit contact activation. The 15 minutes incubation of the sample at 37°C was followed by supplementation with plasminogen activator (TPA, UPA or SK). The sample was recalcified by addition of Ca(CH_3_COO)_2_, 20 mmol/L final concentration, then it was placed into the measuring cuvette. Clotting was initiated with the TF-bearing surface, inserted in the cuvette. Light scattering signal (625 nm) from fibrin clot was captured with a CCD camera.

### Data processing

**Fig 1:**
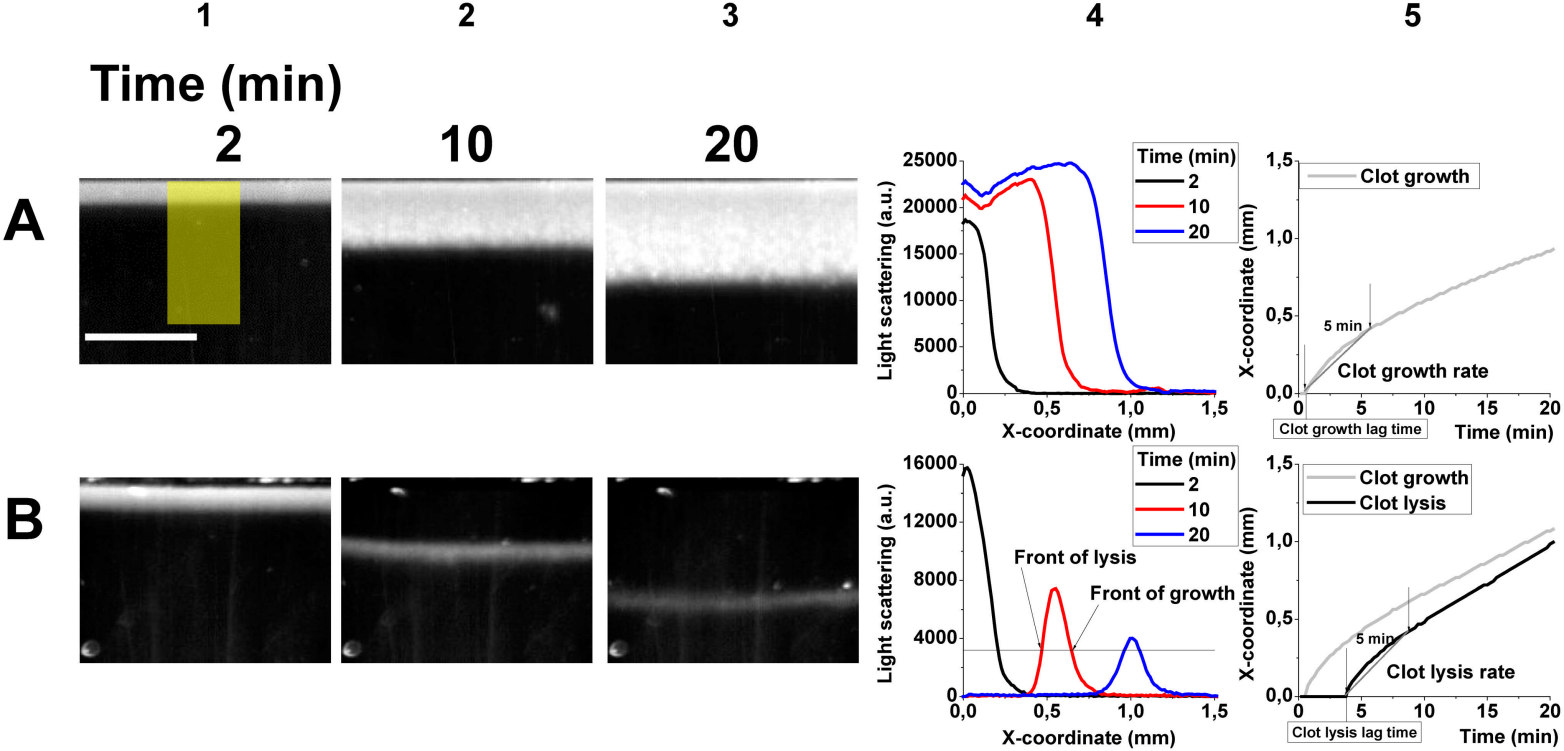
Pictures of fibrin clot growth in the absence of plasminogen activators (2 (A1), 10 (A2) and 20 (A3) minutes after the clotting onset) and clot growth and lysis in the presence of 30 nmol/L of TPA (2 (B1), 10 (B2) and 20 (B3) minutes after the clotting onset). Yellow rectangle on the panel A1 shows the region of the data collection for processing. The scale bar is 1 mm long. Spatial distribution of fibrin in the absence of plasminogen activators (A4) or in the presence of 30 nmol/L of TPA (B4) shows clot propagation and simultaneous clot growth and dissolution, respectively. Black, red, and blue lines show spatial distribution of light scattering signal (proportional to fibrin concentration) at 2^nd^, 10^th^,and 20^th^ minute after initiation of coagulation, respectively. When the signal exceeded the threshold level in any area, we considered that the clot appeared there, and when the level of signal decreased below this threshold, the clot dissolved. Coordinates of these events were designated as fronts of clot growth and lysis. Time course of clot growth front (A5) or clot growth and lysis fronts (B5) allows to calculate the velocities of clot growth or lysis as the average velocity of growth or lysis front propagation within the first 5 minutes after the onset of the process. In order to do that we used its linear approximation within the first 5 minutes after the clotting (lysis) lag time. Clotting (lysis) lag time was calculated as the time when clotting (lysis) front coordinate started to increase.

During the spatial clot growth and lysis experiments, we obtained a set of microphotographs of the fibrin clot (Fig 1). As it was shown previously[23] light scattering was proportional to fibrin concentration; the same linear dependence was established for our experimental setup[24], thus we could treat the intensity of the signal as the relative fibrin concentration. As the light scattering depends also on fiber thickness[25], which in its turn depends on the thrombin concentration, we may assume that in our experimental setup the fibrin clot structure is uniform, due to thrombin which is generated as a propagating wave with peak concentration about 50 nM [11], making light scattering signal depend mainly on fibrin concentration. We averaged the light scattering signal in a horizontal direction (60–80 pixels width) (Fig 1A1, yellow rectangle), obtaining its dependence on the vertical coordinate for each moment of time (Fig 1A4 and 1B4). In order to separate fibrin clot from the liquid plasma, we introduced a threshold level of signal (i.e. fibrin concentration), above which we considered fibrin clot to be formed. We measured the maximal signal value obtained in the experiment, and supposed that the fibrin clot was “formed” at the certain level of the maximal concentration. In all our experiments the maximal signal level was almost the same, so we considered the same amount of fibrin to form the clot. As soon as this threshold was exceeded in any area, we considered that the clot appeared there, and when the level of signal decreased below this threshold, the clot dissolved. We determined the coordinates of these two events for each time step and plotted them over time (Fig 1A5 and 1B5) to estimate the lag time of clotting (lysis) and the velocity of clot growth (lysis). The clotting (lysis) lag time was determined as the time passed since the activation of coagulation up to the moment when the clot growth (lysis) front started to move. Fig 1 5 shows that the velocity of clot growth (lysis) front propagation decreased over time; to characterize it we used its linear approximation within the first 5 minutes after the clotting (lysis) lag time. We compared different threshold levels (10%-50% from maximal signal, Figure A in S1 Appendix) and received very similar results; we chose the threshold level of clot formation as 20% from the maximal level and used it to process all data, both in vitro and in silico.

### Statistical analysis of experimental data

We used Mann-Whitney test with the level of error p = 0.05 to examine the differences in data obtained under different conditions.

### Computational model of fibrin clot growth and lysis

**Fig 2:**
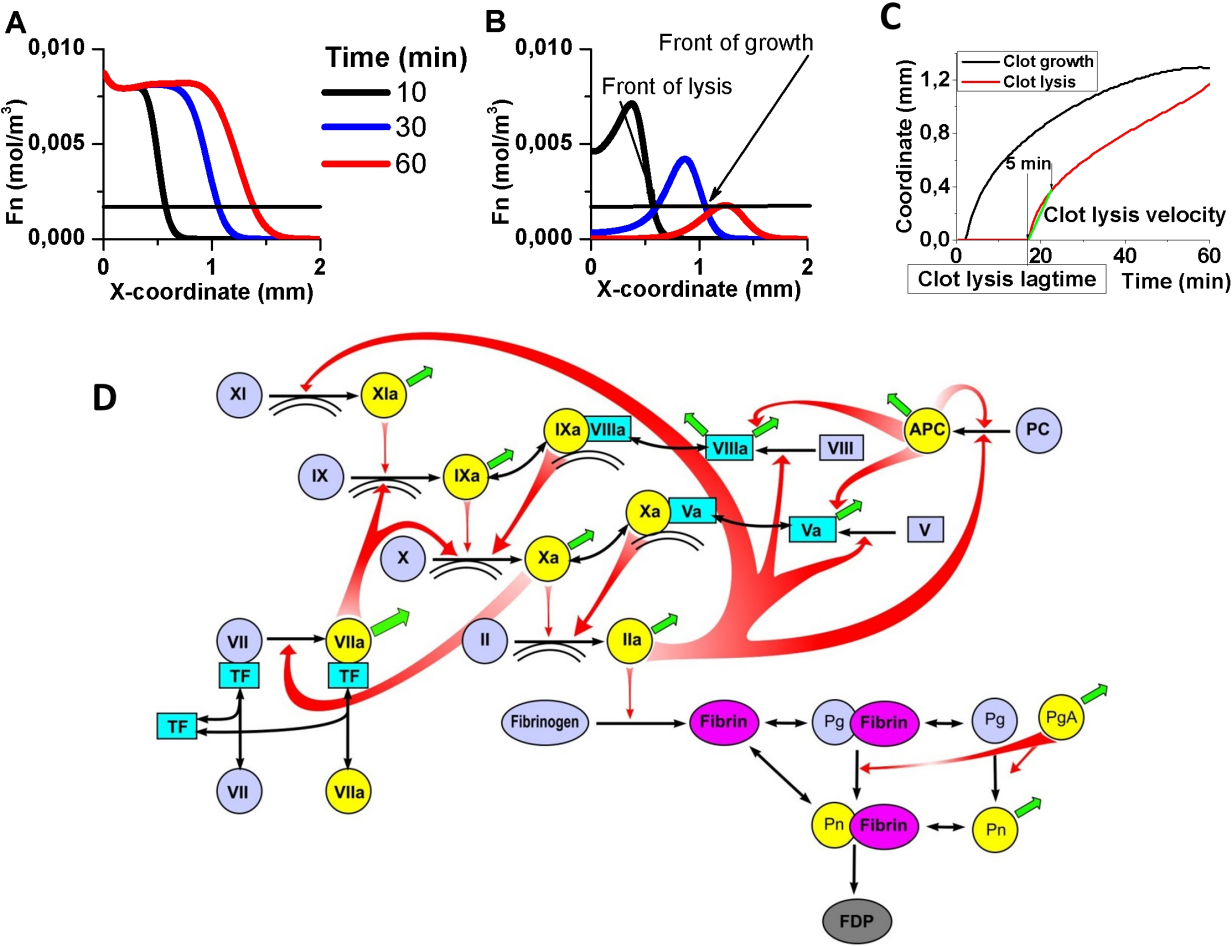
Spatial kinetics of fibrin generation in the absence **(A)** or in the presence **(B)** of 50 nmol/L TPA. Spatial fibrin distribution is shown for 10^th^ (black line), 30^th^ (blue line) and 60^th^ (red line) minute of simulation. **(C)** Time course of clot growth/lysis front during simulation. **(D)** Scheme of blood coagulation cascade main reactions. Zymogens are shown as blue circles, activated proteins are shown as yellow circles. Inactive cofactors are shown as blue rectangles, activated cofactors are shown as cyan rectangles. Red arrows show activation, black arrows show transition from inactive to active form, and formation of complexes. Green arrows show inhibition. Double arc shows phospholipid surface that is required for complex formation or activation. PgA stands for plasminogen activator; FDP stands for fibrin degradation products.

Computational systems biology model of blood clot formation and dissolution was designed to reproduce the experimental design of Fig 1. The model arrangement is shown in Fig 2. Simulations with Comsol 4.3 (Comsol, Burlington, MA) were performed in a 1-dimensional region that was 3 mm long. Clot growth was initiated by tissue factor located at the x = 0. TPA was evenly distributed in the area of simulation (Figure D in S1 Appendix).

Model equations were reaction-diffusion equations based on the laws of Michaelis kinetics and of mass action for the scheme in Fig 2D. A detailed description of the model equations can be found in S1 Appendix (equations S1-S58). Model parameters were mostly from experiments and are given in Tables H, I and J in S1 Appendix.

The "coagulation" part was based on a computational model of one-dimensional spatial clot growth previously developed and validated by our group [17, 18]. Briefly, this part included several distinct modules: a) binding/dissociation of VIIa/VII to TF and feedback activation of VII; b) activation of X and IX by VIIa-TF, c) activation of II by Xa; d) feedback stimulation of V and VIII and XI by thrombin; e) enhanced activation of IX, X, II by XIa, IXa-VIIIa, Xa-Va respectively; f) action of all major stoichiometric inhibitors and negative feedback of the protein C pathway that destroyed active factors Va and VIIIa. The variables of the of the model were total concentrations of factors VIIa, VII, IXa, IX, Xa, X, IIa, II, VIIIa, VIII, Va, V, XIa, XI, zymogen protein C, activated protein C, Xa-TFPI, TFPI, AT-III, fibrinogen, fibrin. To mimic surface activation, we used surface densities of TF, VIIa-TF, and VII-TF, and couples surface reactions with the volume reactions using boundary conditions.

The "fibrinolytic" part of the model is completely novel. The variables include total concentrations of free TPA, plasminogen in the glu- and lys-forms, plasmin, all them in the fibrin-bound form, and also a2M, a2AP, and PAI-1. Fibrinolysis is triggered by binding of plasminogen and TPA to fibrin leading to plasmin formation and fibrin degradation.

The major model assumptions include: a) equlibrium binding of all membrane complexes[26]; b) platelet-derived microparticles and lipoproteins provide most of the membrane surface in plasma[27]; c) no feedback formation of two-chain TPA.; d) fibrin formation and destruction are assumed to be simple one-stage reactions; e) we do not consider Thrombin Activatable Fibrinolysis Inhibitor (TAFI)- and factor XIII-dependent feedforward reactions.

A typical equation of surface reactions for TF-mediated initiation of clotting:

$$\frac{d\sigma_{VII-TF}}{dt}=k_{a}^{VII,TF}\cdot {\left[VII\right]|}_{x=0}\cdot \sigma_{TF}-k_{d}^{VII-TF}\cdot \sigma_{VII-TF}-\frac{k_{cat}^{VII-TF,IIa}\cdot \sigma_{VII-TF}\cdot {\left[IIa^{F}\right]|}_{x=0}}{K_{M}^{VII-TF,IIa}+{\left[IIa^{F}\right]|}_{x=0}}-k_{eff}^{VII-TF,Xa}\cdot \sigma_{VII-TF}\cdot {\left[Xa^{F}\right]|}_{x=0}$$ (1)

On the left, the rate of inactive extrinsic tenase (*σ*_*VII–TF*_) concentration change with time is shown. According to the equation, it is determined by two processes given on the right: 1) association and dissociation of TF with FVII (the first and the second terms); 2) activation of inactive extrinsic tenase by thrombin (the third term) and FXa (the fourth term).

A typical equation of volume reactions:

$$\frac{d\left[IIa\right]}{dt}=D_{IIa}\cdot \frac{\partial^{2}\left[IIa^{F}\right]}{\partial x^{2}}+k_{eff}^{II,Xa}\cdot N_{a}\cdot \left[Xa^{F}\right]\cdot \left[II\right]+\frac{k_{cat}^{localII,Xa-Va}\cdot \left[Xa-Va^{B}\right]\cdot \left[II^{B}\right]}{K_{M}^{localII,Xa-Va}/k\cdot N_{a}}-\left(k_{a}^{IIa,AT-III}\cdot \left[AT-III\right]+k_{a}^{IIa,\alpha_{2}M}\cdot {\left[\alpha_{2}M\right]}_{0}+k_{a}^{IIa,\alpha_{1}AT}\cdot {\left[\alpha_{1}AT\right]}_{0}+k_{a}^{IIa,PCI}\cdot {\left[PCI\right]}_{0}\right)\cdot \left[IIa^{F}\right]$$ (2)

On the left, the rate of thrombin concentration change with time is shown. According to the equation, it is determined by three processes given on the right: 1) diffusion of thrombin (the first term); 2) activation of prothrombin by FXa (the second term) or prothmobinase complex (the third term); 3) inhibition of thrombin by antithrombin, a2-macroglobulin, alpha1-antithrypsin and protein C inhibitor (the fourth member).

Surface reactions generate an inward flux of activated factors, which is set as boundary condition for the volume reactions. Based on the known concentrations of all participants of these reactions at some time-point and on the kinetic constants, we calculate further dynamics of coagulation using a set of equations (S1 Appendix).

## Results

### Spatial lysis of fibrin clot formed in the presence of plasminogen activators starts from the area of clotting activation

**Fig 3:**
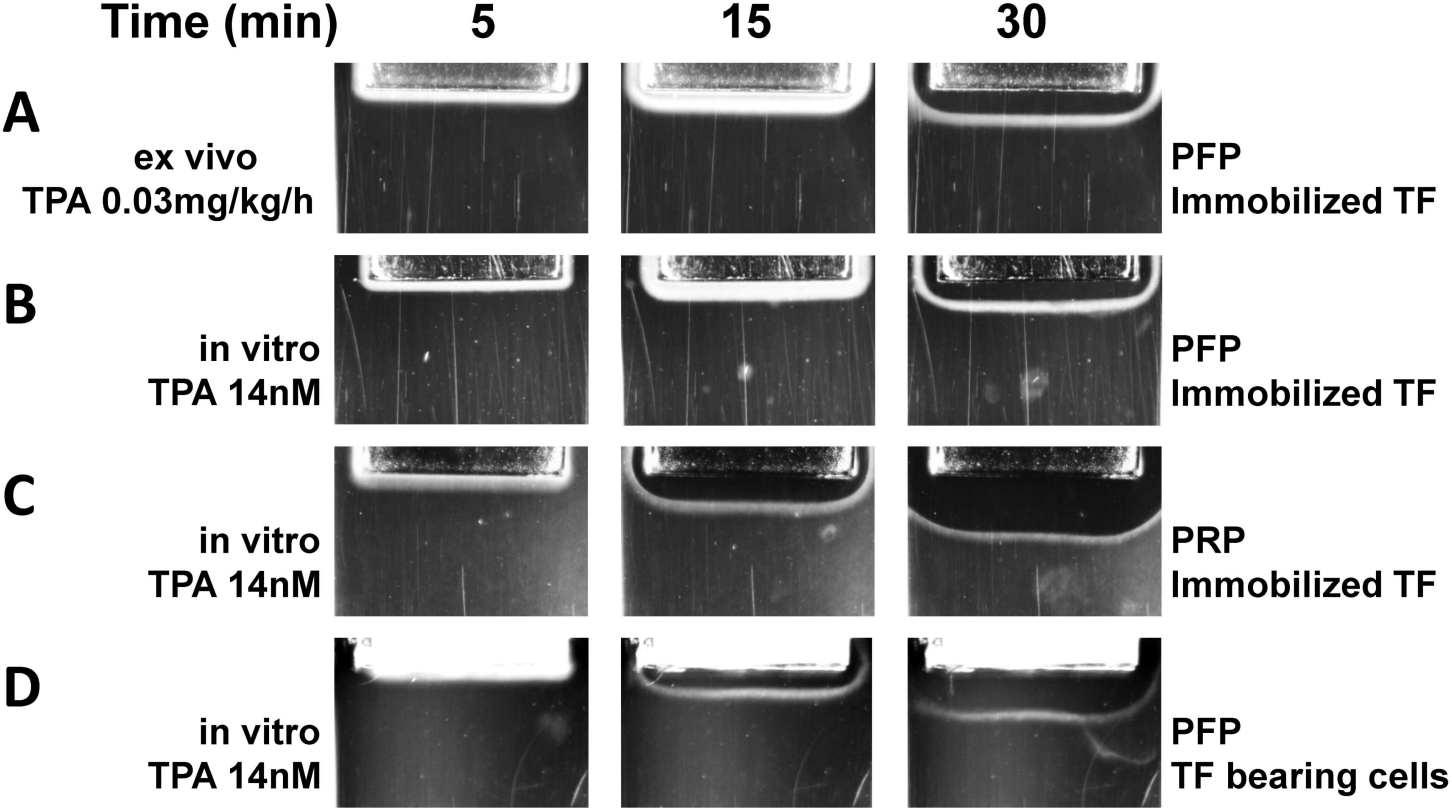
Pictures of fibrin clot growth and lysis in blood plasma 5, 15 and 30 minutes after the start of experiment. Grey rectangle on the top of each image is an inset with immobilized TF. Fibrin clot is white, while liquid plasma is black/dark grey. (A) PFP from a patient under 0.03 mg/kg/h TPA therapy. (B) Pooled PFP from healthy volunteers supplemented with 14 nmol/L TPA in vitro. (C) PRP from healthy volunteer supplemented with 14 nmol/L TPA in vitro. (D) Pooled PFP from healthy volunteers supplemented with 14 nmol/L TPA in vitro, clotting was initiated with a TF-bearing fibroblasts confluent monolayer. Clot growth and lysis were monitored in plasma, treated as described in Methods section. Individual experiments are shown.

Spatial dynamics of clot formation in plasma from healthy volunteers[28,29] or patients with hemophilia A[28] or sepsis[21] showed that no clot lysis occurred within the time of observation (up to 1 hour) in the absence of fibrinolysis activators. Ex vivo spatial clot growth in the PFP from the patient under fibrinolytic therapy with recombinant TPA (Actilyse, Boehringer Ingelheim, Germany) was accompanied by a simultaneous clot lysis: the front of lysis propagation appeared on the clotting activation surface and followed clot growth (S2 Video, Fig 3A).

While previous studies investigated external spatial lysis [30,31], our setup was focused on internal spatial lysis, when clotting and lysis were activated simultaneously. We attempted to obtain this phenomenon in a simple and reproducible model of spatial clot lysis, where we could set any initial conditions. We were able to observe it in PFP from healthy volunteers, supplemented with TPA in vitro (S3 Video, Fig 3B).

In order to investigate whether this phenomenon would persist under more physiological conditions, we also performed these experiments in platelet-rich plasma (PRP, 300,000 platelets/μl) and found the very same pattern of spatial clot lysis (although the velocities of clot growth and lysis propagation were higher in PRP) (S4 Video, Fig 3C). Alternatively, we activated clotting in PFP with a confluent layer of fibroblasts (S5 Video, Fig 3D) and observed the same spatial lysis (also with increased velocities of both clot growth and lysis). Finally, the same result was in PRP where clotting was activated with fibroblasts (Figure B in S1 Appendix, S6 Video). Based on these data, all further research was conducted in PFP with immobilized TF clotting activation, as this setup was the most reproducible and straightforward.

### Characterization of spatial clot growth and lysis

Fibrin clot growth in the absence of plasminogen activator started in the vicinity of the TF-bearing surface and propagated from it in the bulk of plasma (Fig 1A1–1A3). Fig 1A4 shows the dependence of fibrin clot light scattering intensity on the distance from the clotting activating surface. Clot growth is characterized by a steep front of the signal change, which moves in time with the gradually decreasing speed (Fig 1A5). In the presence of 30 nmol/L TPA (Fig 1B1–1B3), clot growth started similarly but was followed by the decrease of the light scattering signal, which started from the same site as the growth did, and propagated in the same direction. We used clotting and lysis lag times (see Materials and Methods) to determine the onset of clotting and lysis; clotting and lysis propagation velocities to determine the initial spatial aspects of clotting and lysis.

### Three regimes of spatial lysis

**Fig 4:**
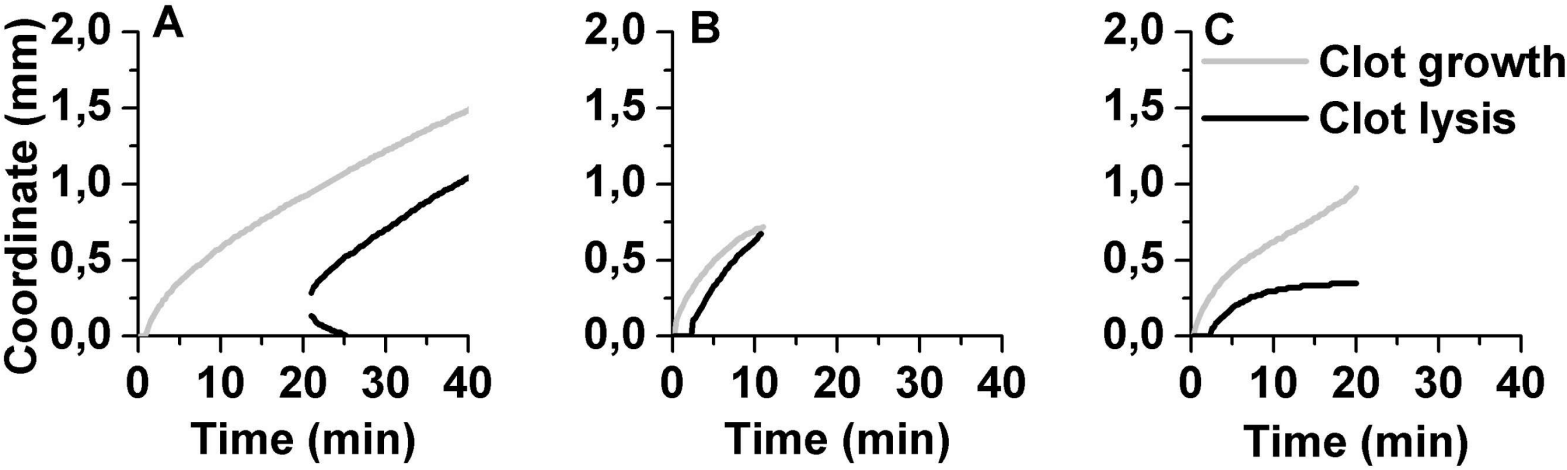
Three regimes of spatial clot lysis. Pooled PFP from healthy volunteers supplemented with increasing concentration of TPA. Clot lysis started 200 μm away from the clotting activating surface in the presence of 6 nmol/L TPA (A); complete clot dissolution was observed in the presence of 100 nmol/L TPA (B); spatial clot lysis stopped 300 μm away from clotting activating surface in the presence of 800 nmol/L TPA (C). Clot growth and lysis were monitored in fresh frozen normal pooled plasma, treated as described in Methods section. Individual experiments are shown.

In order to determine the dose dependent effects of spatial clot lysis parameters, we spiked plasma with increasing concentrations of TPA. We found that TPA dose escalation caused different scenarios of spatial lysis:

1. clot lysis started about 200 μm away from the clotting activator, and 2 fronts of lysis propagated in opposite directions (TPA 6 nmol/L (Fig 4A, Figure C in S1 Appendix)). For 6 nmol/L of TPA the chance of this event was 100% (n = 4), for 14 nmol/L of TPA it was 73% (n = 19), for 30 nmol/L of TPA it was 11% (n = 18); for TPA concentrations higher than 30 nmol/L, clot lysis always started at the TF-bearing surface.
2. clot lysis started at the TF-bearing surface and the lytic front met the clotting front and the clot was completely dissolved (TPA 100 nmol/L (Fig 4B));
3. clot lysis started at the TF-bearing surface and stopped at some distance from it, while the clot growth continued (TPA 800 nmol/L (Fig 4C)).

### Shape of spatial lysis front propagation does not depend on plasminogen activator type

**Fig 5:**
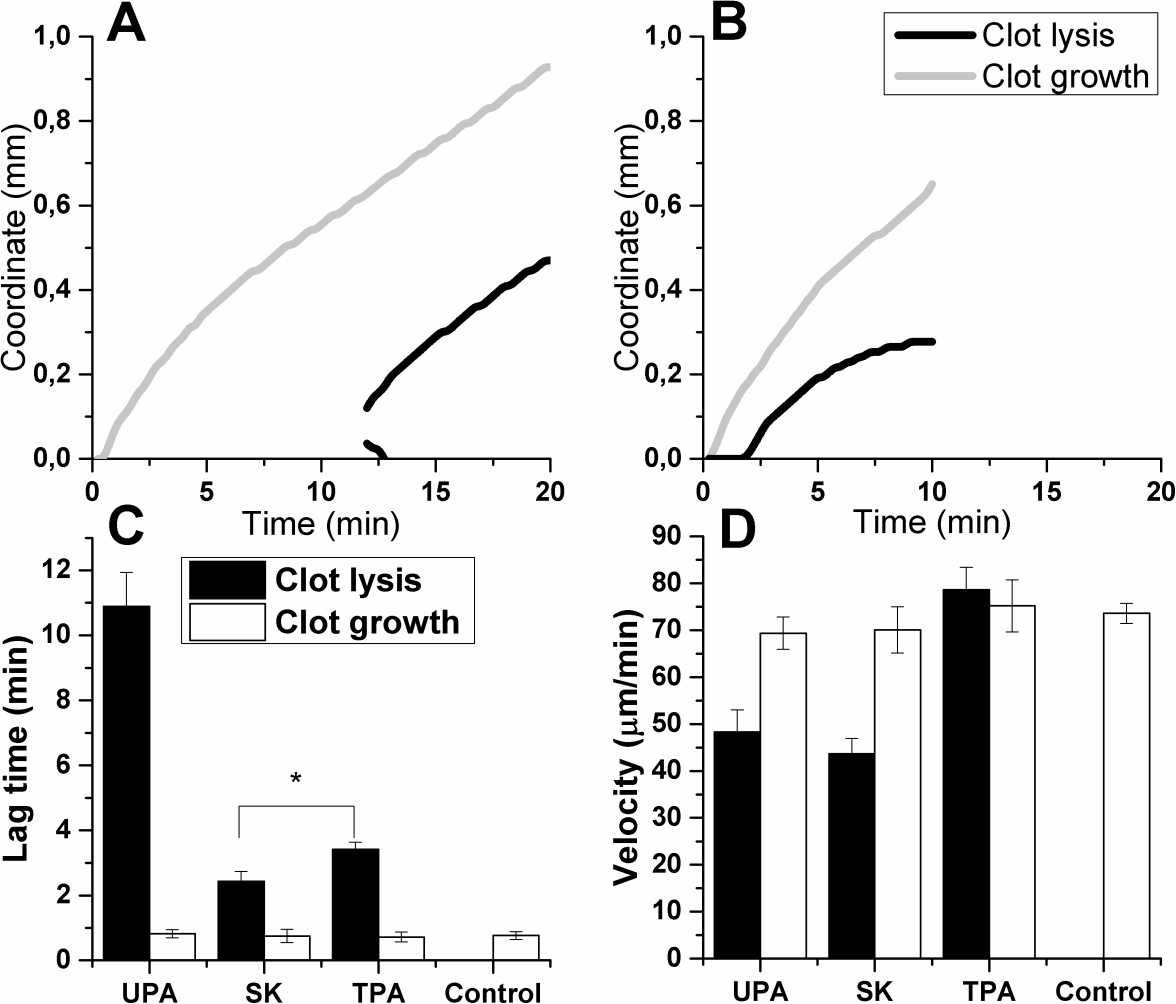
Kinetics of clot growth and lysis fronts coordinate in the presence of 30 nmol/L UPA (A) or 1200 nmol/L SK(B). Spatial clot lysis lag time and velocity depended on the type of plasminogen activator, while spatial clot growth did not change compared to control. The lag times of lysis (C) were significantly different for all plasminogen activators (2.4±0.3 min for SK, 3.4±0.2 min for TPA and 11±1 min for UPA). The velocity of lysis (D) was the same for UPA (48±4 μm/min) and SK (44±3 μm/min), but it was about 1.5 times higher for TPA (78±5 μm/min). Clot growth velocity and lag time were not significantly different from control (74±2 μm/min and 0.77±0.14 min, respectively). Clot growth and lysis were monitored in fresh frozen normal pooled plasma, treated as described in Methods section and supplemented with vehicle (control, n = 7), 50 nmol/L TPA (n = 9), 30 nmol/L UPA (n = 7) or 1200 nmol/L SK (n = 7).

We chose concentration of plasminogen activators to fit the range used during fibrinolytic therapy (TPA 50 nmol/L (60 nmol/L for acute myocardial infarction[32]), UPA 30 nmol/L (180 IU/ml[33]), SK 1200nmol/L (200 IU/ml[34])). The pattern of clot lysis front propagation for UPA (Fig 5A) was similar to the one for low TPA concentration (Fig 4A): lysis started about 100 μm away from the TF-bearing surface and two fronts of lysis propagated from the point of onset, towards the clotting activator and away from it. The single-chain UPA that we used had limited activity towards plasminogen, which can explain this result. The pattern of clot lysis for SK (Fig 5B) was similar to high TPA concentration (Fig 4C): lysis stopped at some distance from the clotting activator while the clot growth continued.

Plasma supplemented with SK or UPA generated fibrin clots just like the control, and there was no significant difference in clot growth velocity or lag time between different types of plasminogen activators (Table 1, Fig 5C & 5D). The lag time of clot lysis was 2.4±0.3 min for SK, 3.4±0.2 min for TPA and 11±1 min for UPA. The velocity of spatial clot lysis was 43.7±3.2 μm/min for SK, 48.4±4.7 μm/min for UPA (no significant difference), but for TPA the velocity was significantly higher: 78.7±4.8 μm/min. Despite some differences in the plasminogen activator’s activities, all of them generated spatial lysis front propagation and not affecting the initiation of clotting.

**Table 1:** Parameters of clot growth and lysis in the absence and presence of thrombolytics.

Lysis activator	Clot growth		Clot lysis	
	Lag time (min)	Velocity (μm/min)	Lag time (min)	Velocity (μm/min)
None	0.8±0.1	73.6±2.1	-	-
UPA (30 nmol/L)	0.8±0.1	69.4±3.5	11±1	48.4±4.7
SK (1200 nmol/L)	0.7±0.2	70.1±4.9	2.4±0.3	43.7±3.2
TPA (6 nmol/L)	0.7±0.1	73.9±4.2	22.3±1.8	55.3±3.4
TPA (50 nmol/L)	0.7±0.1	75.2±5.6	3.4±0.2	78.7±4.8
TPA (200 nmol/L)	0.6±0.1	78±3	2.0±0.2	77.3±4.4
TPA (800 nmol/L)	0.7±0.1	76.6±4.3	2.5±0.4	41.2±5.6

### TPA concentration determines the onset of lysis and its spatial velocity

**Fig 6:**
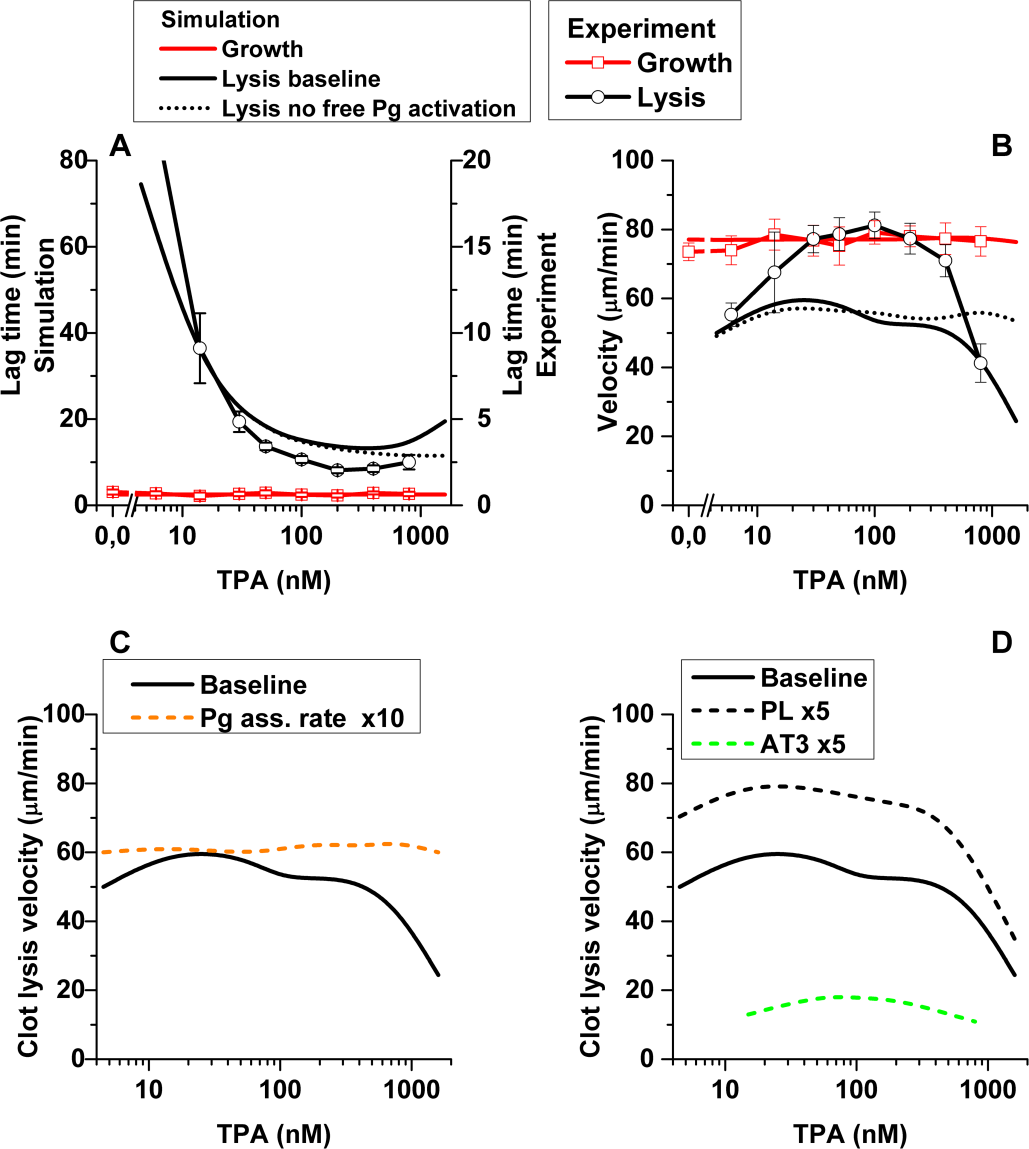
Dependence of clot growth and lysis lag time and velocity on the TPA concentration. (A) Simulation lag time (solid lines) was approximately 4 times higher than experimental lag time (symbols), both for clot growth (red) and clot lysis (black). Lag time of clot growth did not depend on TPA concentration, but lysis lag time decreased with the increase of TPA concentration up to 200 nmol/L, and further increase of TPA caused increase of lag time. (B) Clot growth velocity did not depend on TPA concentration, while lysis velocity was constant (75–78 μm/min) within the TPA concentration range 30–200 nmol/L, and decreased at TPA concentrations >200 nmol/L and <15 nmol/L. In mathematical simulations clot growth velocity was the same as the experimental one; clot lysis velocity was lower than the experimental for TPA concentrations 15–400 nmol/L. Prohibition of free plasminogen activation by TPA (dotted line) removed the increase of lag time and the drop of lysis velocity at high TPA concentrations. (C) Tenfold increase (orange dash line) of the rate of plasminogen association with fibrin made clot lysis velocity insensitive to TPA concentration. Tenfold decrease of the rate of plasminogen association with fibrin cancelled lysis. (D) Changes in plasma procoagulant state, like supplementation with antithrombin III (fivefold increase compared to the baseline, green dash line) or with phospholipids (fivefold increase compared to the baseline, black dash line) decreased or increased respectively the velocity of clot lysis. Clot growth and lysis were monitored in fresh frozen normal pooled plasma (panels A&B), treated as described in Methods section and supplemented with vehicle (control) or TPA at different concentrations (N = 4–11).

The lag time of spatial clot growth did not change with TPA dose escalation (Table 1, Fig 6A). The velocity of clot growth was 73–78 μm/min within the TPA concentration range of 0–800 nmol/L (no significant difference) (Table 1, Fig 6B). Thus, clotting initiation was not affected by TPA.

The lag time of clot lysis decreased from 22±2 min for TPA 6 nmol/L down to 2.0±0.2 min for TPA 200 nmol/L and slightly increased up to 2.5±0.4 min for TPA 800 nmol/L (significantly different) (Fig 6A). The spatial clot lysis velocity increased from 55±3 μm/min for TPA 6 nmol/L up to 75–78 μm/min for the TPA concentration range of 30–200 nmol/L and decreased down to 41±5 μm/min for TPA 800 nmol/L (Fig 6B).

### Computer simulation of spatial clot growth and lysis

Spatial kinetics of fibrin formation in the absence of TPA was similar to the one we observed in vitro (Fig 2). In our simulations, clotting lag time was 2.5 minutes (0.77±0.14 min *in vitro*) and clot growth velocity was 77 μm/min (74±2 μm/min *in vitro*). It may mean that our understanding of clotting initiation that was embodied in the corresponding part of the model requires further improvement. In addition, we need to note that as commercially available immobilized TF was poorly characterized, the exact constants of clotting initiation may differ from that we obtained from literature. However, this discrepancy in simulations and in vitro data had minimal impact on the subject of this work.

We found that spatial clotting in plasma supplemented with plasminogen activator generated two waves that switched plasma from liquid state to solid state and back to liquid (Figure E in S1 Appendix). The wave of thrombin caused fibrin generation which started on the very front of it and gelated plasma. The wave of plasmin caused fibrin degradation which liquefied plasma. The initiation signal (TF) was located on the boundary of the area and these waves were propagating in a self-sustaining manner, which meant that plasminogen activator supplemented plasma was an active medium supporting two different autowave-like modes.

Simulations (Fig 6A & 6B) showed good correlation with the experimental data. Spatial clot lysis lag time decreased from 74.5 min for TPA 4.5 nmol/L down to 13 min for TPA 400 nmol/L and increased up to 19.5 min for TPA 1600 nmol/L (Fig 6A), showing the same dependence of lysis lag time on TPA concentration as *in vitro*. The velocity of spatial clot lysis increased from 50 μm/min for TPA 4.5 nmol/L up to 60 μm/min for TPA 15 nmol/L, slightly decrease within the range of TPA concentration of 15–400 nmol/L. The velocity decreased down to 24 μm/min for TPA 1600 nmol/L. *In silico* simulations described the velocity of spatial lysis well at low and high TPA concentrations but did not show a pronounced increase of the velocity for the medium TPA concentrations as we observed *in vitro*.

We were not able to reproduce the mode of spatial lysis when lysis started about 200 μm away from the TF-bearing surface rather than directly on it; in our simulation lysis always propagated from the clotting activation site.

### The rates of fibrinolytic reactions have a minor influence on the spatial lysis velocity

We investigated the mechanisms governing the velocity of lysis propagation using computer simulations. The following lysis related reactions were implicated: 1) plasminogen activation; 2) fibrin degradation; 3) plasmin association with fibrin; 4) plasmin inhibition; 5) plasminogen association with fibrin. In order to figure out the limiting reactions, we increased and decreased their rates tenfold. The change in the velocity of lysis propagation did not exceed 5% for plasminogen activation (reaction 1), fibrin degradation (reaction 2), plasmin association with fibrin (reaction 3) and plasmin inhibition (reaction 4) (Figure F in S1 Appendix).

Changing the rate of plasminogen association with fibrin, we found that its decrease caused lysis termination (no lysis was observed using our lysis definition within 90 minutes simulation). Increase of this rate (Fig 6C, dashed orange line) made lysis propagation velocity insensitive to the TPA concentration change.

Downregulation of spatial clot lysis (lag time increase and lysis propagation velocity decrease) was observed both *in vitro* and *in silico*. Simulations showed that high TPA concentration could cause plasmin activation all over the area of simulation, causing plasminogen depletion (Figure G in S1 Appendix). It happened because TPA could activate free, not bound to fibrin, plasminogen (both Glu and Lys forms). In 60 minutes of simulation, 50 nmol/L of TPA activated about 10% of plasminogen, while 1600 nmol/L of TPA activated about 98%. Free plasmin was not protected from inhibition by alpha-2-macroglobulin and antiplasmin, so it was inhibited without playing its role in the clot dissolution. When we "switched off" free plasminogen activation by TPA, no lysis lag time increase (Fig 6A, dotted line) or lysis propagation velocity decrease (Fig 6B, dotted line) upon TPA concentration increase was observed.

Within the range of TPA concentration 30-200nmol/L, when it was not limiting fibrin bound plasminogen activation and did not activate free plasminogen enough for its depletion, clot lysis velocity was almost insensitive to any fibrinolysis parameters variation.

### Spatial clot lysis velocity depends on the velocity of fibrin clot propagation

We calculated the dependency of spatial lysis velocity in the system with enhanced coagulation (supplemented with extra phospholipids (PL), clot growth velocity 93 μm/min) (Fig 6D, black dash line) and in the system with suppressed coagulation (supplemented with extra ATIII, clot growth velocity 37 μm/min) (Fig 5D, green dash line). Qualitative dose dependence of spatial lysis velocity on TPA concentration looked the same as the baseline, but the velocity of lysis was higher in the system with PL and lower in the system with ATIII.

**Fig 7:**
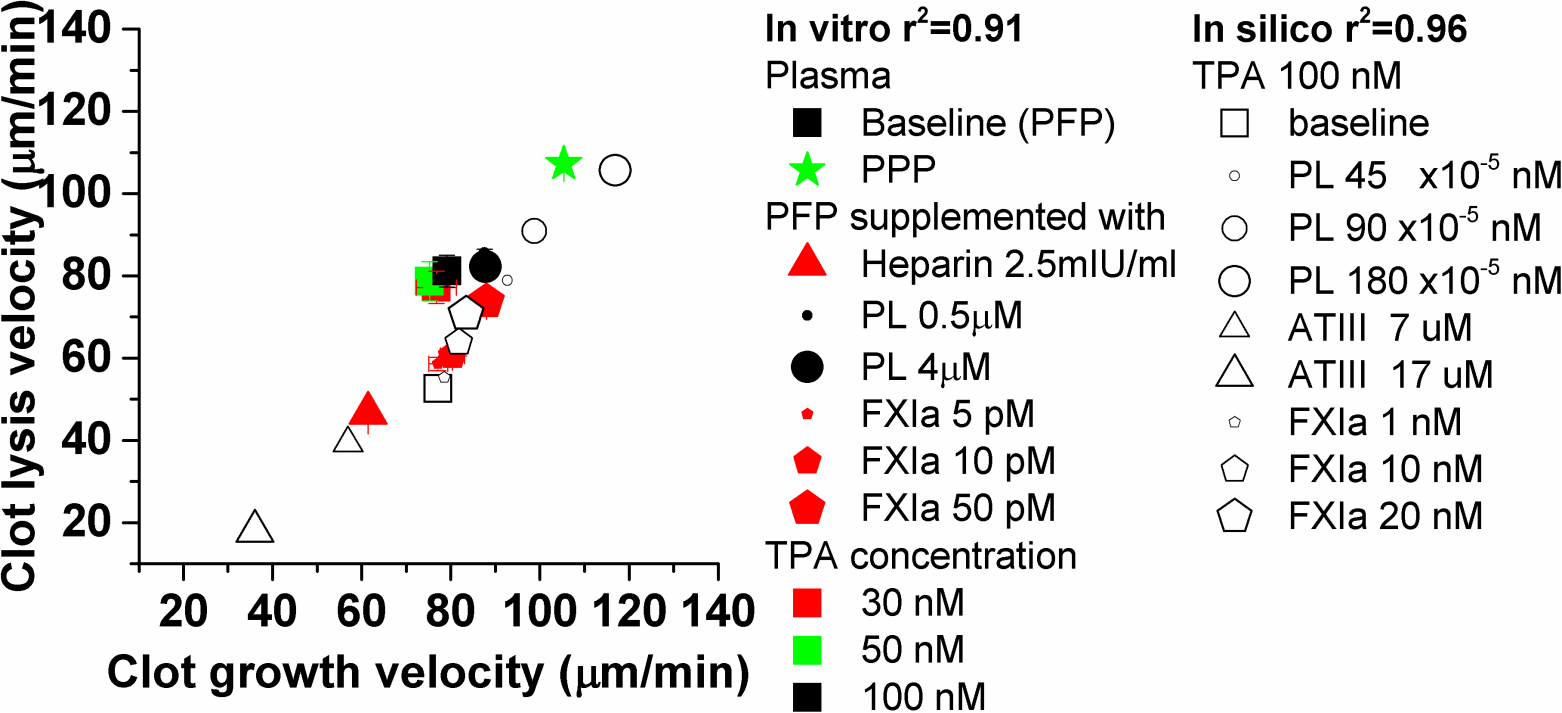
Clot lysis velocity correlated with the clot growth velocity in mathematical simulation (r^2^ = 0.96, opened symbols) and *in vitro* experiments (r^2^ = 0.91, closed symbols, N = 2–6). In simulations plasma was supplemented with 100 nmol/L TPA; 1, 10 or 20 nmol/L FXIa (pentagons) or 45, 90 or 180 x 10^−5^ nmol/L PL (final concentration, circles); 7 or 17 μmol/L ATIII (final concentration, triangles). *In vitro* normal pooled plasma was supplemented with 30 (red), 50 (green) or 100 nmol/L (black) of TPA; 0.5 or 4 μmol/L PL (circles); 5, 10 or 50 pmol/L FXIa (pentagons); 2.5 mU/ml of unfractionated heparin (triangle). Spatial clot lysis in PPP, supplemented with 50 nmol/L TPA had a very high clot lysis velocity and was accompanied by a high clot growth velocity (star). Clot growth and lysis were monitored in plasma, treated as described in Methods section.

We performed *in silico* simulations in plasma (Fig 7) supplemented with 100 nmol/L TPA, where coagulation was enhanced by supplementation with 1–20 nmol/L of FXIa (black pentagons) or 45–180 x 10^−5^ nmol/L of PL (final concentration, black circles), or where coagulation was suppressed by supplementation with 7–17 μmol/L of ATIII (final concentration, black triangles). Clot lysis velocity showed good correlation with clot growth velocity (r^2^ = 0.96).

To support our simulations, we performed *in vitro* experiments in PFP (Fig 7) supplemented with 30 (red), 50 (green) or 100 nmol/L (black) TPA to start lysis; 0.5 or 4 μmol/L PL (closed circles) or 5, 10 or 50 pmol/L FXIa (closed pentagons) to enhance coagulation; 0.0025 IU/ml of unfractionated heparin (closed triangle) to suppress coagulation. In addition, we checked spatial lysis velocity in PPP supplemented with 50 nmol/L of TPA (closed star). We found that the spatial clot lysis velocity correlated with the spatial clot growth velocity (r^2^ = 0.91). Albeit the simulation results did not reproduce the experimental results quantitatively, the qualitative data correspondence was very high. Such dependence of clot lysis velocity on clot growth velocity coupled with insensitivity of clot lysis velocity to almost all fibrinolysis reaction rates (S6 Fig in S1 Appendix) may indicate that only clot formation determines the intrinsic lysis, at least in our experimental conditions.

## Discussion

In this work, we explored the mechanisms of the spatial clot lysis observed in blood plasma, supplemented with the therapeutic concentrations of thrombolytic drugs *ex vivo* and *in vitro*.

As fibrinolytic activity is localized in the fibrin clot, which grows in space over time, two different scenarios of clot interaction with fibrinolytic system are possible: 1) fibrinolysis is initiated when clot contacts thrombolytic, and 2) clot formation and fibrinolysis are activated simultaneously, when thrombolytic supplemented blood plasma contacts clotting activation site. The first scenario describes the extrinsic lysis, removal of life-threatening thrombi during stroke or infarction episodes and is achieved by administration of TPA, SK[35] or UPA[36]. Investigation of thrombolysis mechanisms in such spatial setup[30,31,37,38] allowed to find dependences of lysis on thickness of clot fibers[9,39], shear stress at the moment of clot formation[40], role of different lysis inhibitors[41]. It was shown that lysis started on the outer edge of the clot; the velocity of lysis front propagation increased with the increase of TPA concentration [31]. Computer simulations of clot lysis were engaged to describe the effects of fiber thickness[42], flow velocity[43], spatial lysis propagation [15,43,44]. Here, we investigated the second scenario, intrinsic lysis, which described the physiologic process of clot removal; overstimulation of this process also occurs during thrombolytic therapy.

We found that the front of lysis appeared on the clotting activating surface and propagated in the same direction as the front of clot growth, detaching the forming clot from the TF-bearing surface. Albeit in the most of our experiments we used PFP and immobilized TF, we consider that our findings can be used to estimate the *in vivo* process, as the manifestation of the spatial lysis did not change in PRP and/or when clotting was activated with TF-bearing cells. We used fetal lung fibroblasts which release UPA at very low level [45]. Also, UPA is secreted as a single-chain proenzyme (pro-uPA) that possesses little or no proteolytic activity. As in our experimental setup we used TPA, which single-chain form has equal activity to the two-chain form when fibrin bound [46], we can neglect the fibroblast released UPA contribution in the process of clot lysis.

Phenomenon of spatial lysis was also observed in plasma of patient under fibrinolytic therapy, when no plasminogen activators were added to plasma in vitro. This experiment can be a proof-of-principle demonstrating that concentration of plasminogen activators during thrombolytic therapy can be high enough to cause intrinsic lysis. Further investigation in plasma of patients under fibrinolytic therapy is needed to estimate the clinical relevance of the spatial clot lysis.

In our experiments we used a wide range of supplemented TPA concentrations, from 0 up to 800 nM. This range is much beyond the TPA level of 70pM which corresponds to intact plasma. After thrombosis induction fibrinolytic activity of plasma increases in 20 times [47]. One of the mechanisms responsible for that is thrombin mediated TPA release from endothelial cells [48]. TPA local concentration in the vicinity of endothelial wall (where the clot forms) is rather high, and we assume that it might reach tens of nM. Besides that, very high TPA concentration can be reached in blood plasma during thrombolytic therapy. During a double bolus regimen of TPA administration (patients with AMI who received systemic thrombolysis with alteplase by the double bolus regimen, consisting of the administration of two boluses of 50 mg at an interval of 30 minutes) [49] TPA peak concentration may reach 200-300nM (estimation, based on average blood volume and 100% bioavailability of TPA). In order to better understand the mechanisms of fibrinolysis and blood coagulation system interaction we increased the range of TPA concentration up to 800 nM.

Also, we showed that high concentration of plasminogen activator caused lysis termination in the proximity of the clotting activator. Computer simulation of spatial lysis revealed that it was caused by plasminogen depletion, when free plasminogen was activated by free TPA and then plasmin which was not protected by fibrin was inhibited without playing its role in clot dissolution. This finding was indirectly confirmed by our experiment with SK, a non-fibrin-specific fibrinolytic agent, which activated plasminogen both free and fibrin-bound[50], and by the previous findings when high concentrations of TPA led to the impairment of clot lysis[51]. Our setup let us figure out that in this case lysis was impaired in a very specific manner, it detached clot from the activating surface and then stopped its propagation. This regime of clot lysis can potentially be dangerous for the patient, as it may cause emboli formation or rethrombosis, leading to the necessity of the therapy monitoring by means of clot spatial dynamics.

Analysis of our computer simulations showed that spatial lysis velocity did not depend on the rates of plasminogen interactions with its activators, but it was mostly determined by the velocity of clot growth, and it was experimentally confirmed when we found good correlation of the spatial lysis velocity and the clot growth velocity. Thus, the overall manifestation of the spatial lysis can be explained when we take a closer look on the mechanism of clot growth. Clot growth is caused by an impulse-like wave of thrombin propagation[11] with the constant peak height that generates a fibrin clot with a uniform structure. Plasmin generation starts on the fibrin clot as soon as it appears, causing dissolution of the clot near the clotting activator earlier than the clot farther away. Thus, plasminogen activator supplemented blood plasma represents an active medium that supports two waves, which switches its state from liquid to solid (thrombin wave) and then back to liquid (plasmin wave).

Our computer simulations were not able to reproduce clot lysis pattern observed at low TPA concentrations, when the clot dissolution started in some distance away from the clotting activation site. We propose the following explanation for this phenomenon. Distribution of thrombin concentration during the clot formation is very high within 150–200 μm from the clotting activator[11], and then it changes in space and time as a traveling wave with almost constant peak height. High concentrations of thrombin in the vicinity of the clotting activator form the denser clot (it was shown for fibroblast cells[8,52]), which is more resistant to lysis when the activation signal is low. High thrombin concentration can activate more FXIII, which can make the fibrin clot more resilient to lysis, or it can activate more TAFI, which can prevent plasmin binding to fibrin thus delaying lysis. Further incorporation of these factors coupled with the *in vitro* experiments in FXIII- and TAFI-deficient plasmas could be helpful for the understanding of spatial clot lysis.

Speculating about the relevance of spatial clot lysis propagation, we may assume the following scenario. In spite of low TPA half-life (less than 5 minutes[53]), continuous infusions (from 3h for acute myocardial infarction[54] up to 24–36 h in acute limb ischemia and DVT[55,56]) may supply high concentrations of circulating TPA long enough to develop the spatial lysis pattern we found in our work. Any clot formation during a thrombolytic therapy may be accompanied by the simultaneous clot lysis with the possibility of the clot detachment from the procoagulant surface. The detached clot may completely dissolve, but at the certain conditions it may become a part of an embolus, which can explain such side effects of thrombolytic therapy as rethrombosis[57] or thromboembolism[58,59]. The danger of these effects may be highly increased in the patients with hypercoagulation. We may also assume that the excess of plasminogen activators in blood during therapy may cause plasminogen depletion, which increases the chances of a detached clot to survive and contribute in an adverse event. Thus, the use of global hemostasis assays for continuous monitoring of the blood coagulation state of a patient under fibrinolytic therapy in order to make the corresponding corrections to the hemostasis may be helpful in reducing the risk of adverse events.

## Supporting information

S1 Appendix Supplementary information. Contains supporting data and detailed description of mathematical model of blood plasma coagulation and fibrinolysis.
(DOCX)

S1 Video Ex vivo, no plasminogen activator. Spatial clot growth videomicroscopy assay prior to the thrombolytic therapy was started. Time scale is 1:150 (1s of video corresponds to 150 s of experiment). Recorded length of experiment is 30 min.
(AVI)

S2 Video Ex vivo, after tissue plasminogen activator was administrated. Spatial clot growth videomicroscopy assay after the thrombolytic therapy was started. Time scale is 1:150 (1s of video corresponds to 150 s of experiment). Recorded length of experiment is 30 min.
(AVI)

S3 Video Platelet free plasma supplemented with TPA in vitro. Clotting was activated with immobilized TF. Time scale is 1:150 (1s of video corresponds to 150 s of experiment). Recorded length of experiment is 30 min.
(AVI)

S4 Video Platelet rich plasma supplemented with TPA in vitro. Clotting was activated with immobilized TF. Time scale is 1:150 (1s of video corresponds to 150 s of experiment). Recorded length of experiment is 30 min.
(AVI)

S5 Video Platelet free plasma supplemented with TPA in vitro. Clotting was activated with confluent layer of fibroblasts. Time scale is 1:150 (1s of video corresponds to 150 s of experiment). Recorded length of experiment is 30 min.
(AVI)

S6 Video Platelet rich plasma supplemented with TPA in vitro. Clotting was activated with confluent layer of fibroblasts. Time scale is 1:150 (1s of video corresponds to 150 s of experiment). Recorded length of experiment is 30 min.
(AVI)
