# Supplementary material for: Co-ordinated spatial propagation of blood plasma clotting and fibrinolytic fronts
Source: PLoS One. 2017 Jul 7;12(7):e0180668. doi: 10.1371/journal.pone.0180668 (PMC5501595; doi:10.1371/journal.pone.0180668)
Supplement: S1 Appendix — Contains supporting data and detailed description of mathematical model of blood plasma coagulation and fibrinolysis. (DOCX) [file pone.0180668.s001.docx]

**Title:** Co-ordinated spatial propagation of blood plasma clotting and fibrinolytic waves

**Authors:** Ansar S. Zhalyalov, Mikhail A. Panteleev, Marina A. Gracheva, Fazoil I. Ataullakhanov, Alexey M. Shibeko.

**Supplementary information**

Contents

[Patient 2](#_Toc485933353)

[Spatial lysis in vitro 3](#_Toc485933354)

[Fig. A. Dependence of spatial clotting and lysis parameters on threshold level of clot gelation. 3](#_Toc485933355)

[Fig. B. Pictures of fibrin clot growth and lysis in platelet rich plasma in the presence of 14 nmol/L TPA. 4](#_Toc485933356)

[Fig. C Pictures of fibrin clot growth and lysis in platelet free plasma in the presence of 6 nmol/L TPA. 4](#_Toc485933357)

[Mathematical simulation 5](#_Toc485933358)

[Fig. D. Area of simulation. 5](#_Toc485933359)

[Fig. E. Two switching waves in plasminogen activator supplemented plasma. 6](#_Toc485933360)

[Fig. F. Simulation of spatial clot lysis. 7](#_Toc485933361)

[Fig. G. Downregulation of spatial lysis rate by plasminogen depletion. 8](#_Toc485933362)

[Mathematical model description 9](#_Toc485933363)

[Equations: 9](#_Toc485933364)

[Table H. Model parameters: initial conditions 16](#_Toc485933365)

[Table I. Model parameters: kinetic constants. 17](#_Toc485933366)

[Table J. Model parameters: diffusion coefficients 21](#_Toc485933367)

# Patient

A 1.5 years old male suffered juvenile myelomonocytic leukemia, MTHFR and PAI-1 heterozygous thrombophilia mutations from the Federal Research and Clinical Centre of Pediatric Hematology, Oncology and Immunology was enrolled in this study and monitored before and after the thrombolytic therapy started. The clinical protocol was approved by the ethics committee of the Federal Research and Clinical Centre of Pediatric Hematology, Oncology and Immunology. The patient had a history of two thrombotic episodes before he started treatment at the Federal Research and Clinical Centre of Pediatric Hematology, Oncology and Immunology.

Patient developed a thrombosis in the left leg, which was diagnosed with the ultrasound. The patient was on the antithrombotic heparin therapy (12.5-20 IU/kg/h). No clot lysis was observed in the spatial clot growth videomicroscopy assay (Video S1). Ultrasound showed the progressing deposition of thrombus near the catheter so the systemic thrombolytic therapy was started (actilyse 0.03 mg/kg/h; heparin 10 IU/kg/h). Next day the left thigh diameter decreased by 2.5 cm and blood circulation normalized. Spatial clot growth assay showed clot lysis (Video S2).

# Spatial lysis in vitro

Fig. A. Dependence of spatial clotting and lysis parameters on threshold level of clot gelation. We used different threshold levels (from 10% up to 50% of maximal signal) to separate solid clot from liquid plasma, and calculated parameters of clotting and lysis for these levels. Clot growth rate (A) was insensitive to the level of clot gelation; clot lysis rate (B) gradually increased with the increase of the level of gelation, but the overall dependence on the TPA concentration look very similar for all values of the threshold. Clot growth lag time (C) increased with the increase of the clotting threshold, while clot lysis lag time almost did not change (D). Based on these observations, we chose 20% of maximal signal as the clotting threshold and used it for all data processing.


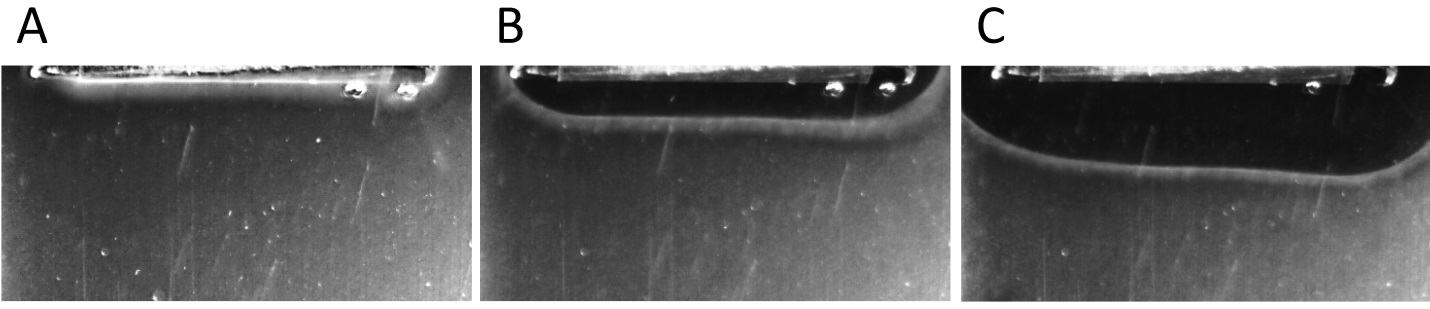


Fig. B. Pictures of fibrin clot growth and lysis in platelet rich plasma in the presence of 14 nmol/L TPA. Pictures of fibrin clot growth and lysis in platelet rich plasma. 5(A), 15(B) and 30(C) minutes after the start of experiment. Clot growth was initiated by a confluent layer of TF-bearing fibroblasts.


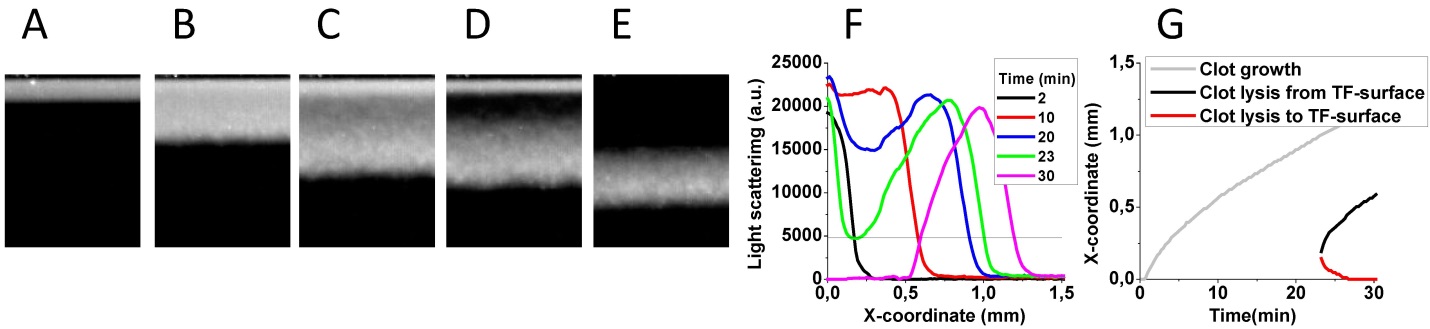


Fig. C Pictures of fibrin clot growth and lysis in platelet free plasma in the presence of 6 nmol/L TPA. 2 (A), 10 (B), 20 (C), 23 (D) and 30 (E) minutes after the clotting onset. Spatial kinetics of fibrin generation shows that clot lysis started about 200 μm from the TF-bearing surface (F), and propagated in both directions, from (black line) and towards (red line) the TF-bearing surface (E).

# Mathematical simulation

Mathematical simulations took place in one-dimensional area 3 mm long, with the mesh of 300 nodes. Clotting activator, tissue factor (TF) was located at the x=0 coordinate, while lysis activator, tissue plasminogen activator (TPA) was distributed evenly all over the area of simulation.

Fig. D. Area of simulation. Coagulation activator (TF) was located at the point x=0, while lysis activator (TPA) was located all over the area of simulation.

Fig. E. Two switching waves in plasminogen activator supplemented plasma. Mathematical simulation of clot growth and lysis was performed in the presence of 100nmol/L TPA and 180∙10^-5^ nmol/L phospholipids. Spatial distribution of thrombin (black), fibrin (red) and plasmin (green) is shown at 10^th^ (panels A, D, G), 20^th^ (panels B, E, H) and 30^th^ (panels C, F, I) minutes of simulation. Panels A, B and C show concentration of thrombin, fibrin and plasmin in nmol/L; thrombin and plasmin concentrations are much lower than fibrin. Panels D, E and F show normalized to maximal value concentrations of thrombin, fibrin and plasmin. As even very low concentration of thrombin can cause clot formation, and the most part of thrombin is generated after clot formation, it looks like the wave of thrombin follows the fibrin propagation. But at the scale of 10 nmol/L (panels G, H and I show thrombin and plasmin in nmol/L) we can see that clotted state of plasma (red rectangular area) follows thrombin wave propagation and clotted state switches back to the unclotted (white) after the wave of plasmin passed.

Investigating the mechanisms governing the rate of spatial lysis, we changed the rates of the following reactions, which were implicated in clot lysis: 1) fibrin degradation; 2) plasmin association with fibrin; 3) plasmin inhibition. We found that 10 times increase or decrease of these rates did not change the rate of spatial lysis more than 3% from the baseline (Fig. S8).

Fig. F. Simulation of spatial clot lysis. Ten times increase or decrease of the rate of the following reactions: 1) plasminogen activation (red – ten times increase; pink – ten times decrease); 2) fibrin degradation (green – ten times increase; yellow – ten times decrease); 3) plasmin association (blue – ten times increase; dark yellow – ten times decrease); 4) plasmin inhibition (cyan – ten times increase; dark blue – ten times decrease) did not change spatial clot lysis rate.

Fig. G. Downregulation of spatial lysis rate by plasminogen depletion. Spatial kinetics of fibrin generation and plasminogen consumption in the presence of (A)TPA 50 nmol/L; (B)TPA 1600 nmol/L. Consumption of plasminogen by free TPA causes the downregulation of the lysis rate.

# Mathematical model description

*Notation*. The concentration of a factor F is denoted [*F*]. All model variables represent total concentrations: e.g., [*IXa*] is the concentration of total IXa including fIXa free in solution, fIXa in complex with fVIIIa, fIXa in the enzyme-substrate complex with fX, etc. The concentration of free factor F in solution is [*F^F^*]; for most factors, [*F^F^*]~[*F*]. The concentration of factor F bound to activated platelets is denoted [*F^B^*];  stands for factor F, which is bound to activated platelets but is free, i.e., not in complex with other factors on platelet membrane. Exception to this notation is , which represents fVa bound to both intact and activated platelets and susceptible to inactivation by APC.

Rate constants of the F_1_ and F_2_ association and dissociation are $k_{a}^{F_{1}, F_{2}}$ and $k_{d}^{F_{1}, F_{2}}$, respectively; equilibrium dissociation constant is designated $K_{d}^{F_{1}, F_{2}}$. Subscript *i* identifies competitive inhibition constant. $K_{d}^{F}$ and $n^{F}$ are the equilibrium dissociation constant and the number of binding sites per platelet, respectively, for factor F. Constants $k_{cat}^{F_{1}, F_{2}}$, $K_{M}^{F_{1}, F_{2}}$, and $k_{eff}^{F_{1}, F_{2}}$ are catalytic, Michaelis and effective rate constants, respectively, for factor F_1_ catalysis by enzyme F_2_. Superscript "local" indicates an intrinsic, two-dimensional constant for reactions on the activated platelet membrane. Symbol *k* in the denominator of local constants is the parameter used to link them with three-dimensional concentrations (1). Symbol $\rho^{F}$ represents the number of molecules of a factor F secreted per platelet upon activation. Surface densities of the factors on the activating surface are designated via σ; for example, σ*_TF_* is the TF surface density. Diffusion coefficient of a factor F is denoted *D_F_*.

## Equations:

**1. Initiation:**

= + +

(S1)

+ –

– –

= – –

(S2)

–

= – –

(S3)

–

= +

(S4)

= –

(S5)

**2. Cascade backbone:**

= + +

(S6)

+ –

= – ––

(S7)

= + +

(S8)

+ –

– –

– –

= – –

(S9)

–

= + + –

(S10)

–

= – –

(S11)

= –

(S12)

= –

(S13)

**3. Cofactor activation:**

$\frac{\partial\left[VIIIa\right]}{\partial t}$=$D_{VIIIa}\cdot\frac{\partial^{2}\left[VIIIa\right]}{\partial x^{2}}$ + $\frac{k_{cat}^{VIII, IIa}\cdot\left[VIII\right]\cdot\left[IIa^{F}\right]}{K_{M}^{VIII, IIa}+\left[IIa^{F}\right]}$ – $h^{VIIIa}\cdot\left[VIIIa\right]$

= –

(S14)

(S15)

= + –

(S16)

– –

= –

(S17)

**4. Inhibition:**

= + + –

(S18)

–

= – –

(S19)

= +

(S20)

= –

(S21)

= – –

(S22)

– $k_{a}^{Xa, AT-III}\cdot\left[AT-III\right]\cdot\left[Xa^{F}\right]$ – $k_{a}^{Xa-Va^{B}, AT-III}\cdot\left[AT-III\right]\cdot\left[Xa-Va^{B}\right]$ –

– $k_{a}^{IIa, AT-III}\cdot\left[AT-III\right]\cdot\left[IIa^{F}\right]$ – $k_{a}^{XIa, AT-III}\cdot\left[AT-III\right]\cdot\left[XIa\right]$ – $k_{a}^{IIa, AT-III}\cdot\left[AT-III\right]\cdot\left[IIa\right]$

**5. Long-range feedback:**

= + –

(S23)

= –

(S24)

**6. Lysis**

= – – +

(S25)

+

(S26)

= –

= – – + –

(S27)

= + – + – –

(S28)

= – – ––

(S29)

= – + +–

(S30)

= + +– + – – +

(S31)

= – – +

(S32)

+–

= –

(S33)

= –– – –

(S34)

= –– –

(S35)

7. The compact notation for free and bound factors and factor complexes concentrations in the sections 1-6 above should be read as follows:

=

(S36)

=

(S37)

=

(S38)

=

(S39)

=

(S40)

[*Xa–Va^B^*]= $\frac{\left[Xa\right]\cdot\left[Va^{B}\right]}{K_{d}^{Xa-Va}\cdot\left(1+\frac{\left[PS\right]}{K_{i}^{Va, PS}}+\frac{\left[Xa\right]}{K_{d}^{Xa-Va}}\right)+\left[Va^{B}\right]}$

(S42)

(S41)

[*Xa^F^*]=[*Xa*] – [*Xa–Va^B^*]

=

(S43)

[*IIa^F^*]=$\frac{\left[IIa\right]}{1+\frac{\left[Fg\right]+\left[Fn\right]}{K_{M}^{Fg, IIa}}}$

(S44)

[*II^B^*]=$\frac{\left[II\right]\cdot N_{a}\cdot n^{X\left(II\right)}}{K_{d}^{II}\cdot\left(1+\frac{\left[X\right]}{K_{d}^{X}}+\frac{\left[II\right]}{K_{d}^{II}}\right)}$

(S45)

[*Va^B^*]=$\frac{\left[Va\right]\cdot N_{a}\cdot n^{Va}}{K_{d}^{Va}+\left[Va\right]}$

(S46)

$\frac{\left[Va\right]\cdot N_{a}\cdot n^{Va}}{K_{d}^{Va}+\left[Va\right]}$– – [*Xa–Va^B^*]

(S48)

(S47)

$$\frac{\left[PS\right]\cdot\left[Va^{B}\right]}{K_{i}^{Va, PS}\cdot\left(1+\frac{\left[PS\right]}{K_{i}^{Va, PS}}+\frac{\left[Xa\right]}{K_{d}^{Xa-Va}}\right)}$$

10. Boundary conditions:

For most factors, non-permeability conditions were used on both ends of the simulation interval. An example is given for prothrombin:

(S49)

(S50)

Exceptions are fIXa, fIX, fXa, fX, Xa-TFPI, TFPI, because the activation reactions occurring on the left end of the simulated interval cause changes in the boundary conditions corresponding to the "flow" of these factors across the boundary:

(S51)

(S52)

(S53)

(S54)

(S55)

(S56)

(S57)

(S58)

It should be noted that for those factors, which have diffusion terms including free factors (factors Xa and IIa), the boundary conditions should be also written for the free factors (for Xa^F^ and IIa^F^).

## Table H. Model parameters: initial conditions

| 1. Initial concentrations of model variables | | | | | |
| --- | --- | --- | --- | --- | --- |
| Factor | Surface density,  moles/m^2^ | Factor | Concentration, nmol/L | Factor | Concentration, nmol/L |
| $\sigma_{VIIa-TF}$ | 0 | VIIa | 0.1 | Va | 0 |
| $\sigma_{VII-TF}$ | 0 | VII | 10 | V | 20 |
| $\sigma_{TF}$ | 5·10^-11^ | IXa | 0 | APC | 0 |
|  |  | IX | 90 | PC | 60 |
|  |  | Xa | 0 | Xa–TFPI | 0 |
|  |  | X | 170 | TFPI | 2.5 |
|  |  | IIa | 0 | AT-III | 3400 |
|  |  | II | 1400 | XIa | 0 |
|  |  | Fn | 0 | XI | 30 |
|  |  | Fg | 7600 | Pg_L | 0 |
|  |  | α_2_AP | 1100 | Pn | 0 |
|  |  | α_2_M | 3000 | TPA | 0-800 |
|  |  | PAI1 | 2 | VIIIa | 0 |
|  |  | Pg_G | 2000 | VIII | 0.7 |
| 2. Constant concentrations of the model | | | | | |
| Factor | Concentration, nmol/L | Factor | Concentration, nmol/L | Factor | Concentration, nmol/L |
| PS | 346 | α_1_AT | 40000 | PCI | 88 |
| N*_a_* | 7.5·10^-5^ | C1I | 1700 |  |  |

## Table I. Model parameters: kinetic constants.

| Constant | Value | Reference |
| --- | --- | --- |
|  | 1. Initiation of coagulation |  |
| $k_{a}^{VIIa, TF}$, $k_{d}^{VIIa-TF}$ | 2.75 nM^-1^min^-1^, 1.1 min^-1^ | (1;2)* |
| $k_{a}^{VII, TF}$, $k_{d}^{VII-TF}$ | 2.75 nM^-1^min^-1^, 1.1 min^-1^ | (1;2)* |
| $k_{cat}^{VII-TF, IIa}$, $K_{M}^{VII-TF, IIa}$ | 3.66 min^-1^, 2700 nM | (3) |
| $k_{eff}^{VII-TF, Xa}$ | 0.4 nM^-1^min^-1^ | (4) |
| $k_{cat}^{VII, IIa}$, $K_{M}^{VII, IIa}$ | 3.66 min^-1^, 2700 nM | (3) |
|  | 2. Cascade backbone |  |
| $k_{cat}^{IX, VIIa-TF}$, $K_{M}^{IX, VIIa-TF}$ | 6.8 min^-1^, 250 nM | (5) |
| $k_{d}^{Xa-VIIa-TF}$ | 770 min^-1^ | (6) |
| $k_{cat}^{IX, XIa}$, $K_{M}^{IX, XIa}$ | 5.8 min^-1^, 200 nM | (7) |
| $k_{cat}^{X, VIIa-TF}$, $K_{M}^{X, VIIa-TF}$ | 40 min^-1^, 390 nM | (5;8;9) |
| $k_{cat}^{X, IXa local}$, $K_{M}^{X, IXa local}/k$ | 0.6 min^-1^, 230 molecules/platelet | (10;11)* |
| $k_{cat}^{X, IXa-VIIIa local}$,  $K_{M}^{X, IXa-VIIIa local}/k$,  $K_{d}^{IXa-VIIIa local}/k$, $K_{d}^{VIIIa-X local}/k$ | 6350 min^-1^,  1216 molecules/platelet,  278 molecules/platelet,  1655 molecules/platelet | (12) |
| $k_{eff }^{II, Xa}$ | 45 nM^-2^min^-1^ | (13;14)* |
| $\frac{k_{cat}^{local II, Xa-Va}}{K_{M}^{local II, Xa-Va}/k}$ | 0.047 min^-1^ | (13)* |
| $k_{cat}^{Fg, IIa}$, $K_{M}^{Fg, IIa}$ | 5040 min^-1^, 7200 nM | (15) |
|  | 3. Cofactor activation |  |
| $k_{cat}^{VIII, IIa}$, $K_{M}^{VIII, IIa}$ | 54 min^-1^, 147 nM | (16) |
| $k_{cat}^{V, IIa}$, $K_{M}^{V, IIa}$ | 14 min^-1^, 71.7 nM | (17) |
|  | 4. Inhibition |  |
| $k_{a}^{VIIa-TF, Xa-TFPI}$ | 0.44 nM^-1^min^-1^ | (18) |
| $k_{a}^{Xa-VIIa-TF, TFPI}$ | 6 nM^-1^min^-1^ | (6) |
| $k_{a}^{Xa, TFPI}$, $k_{d}^{Xa-TFPI}$ | 0.052 nM^-1^min^-1^, 0.02 min^-1^ | (18) |
| $k_{a}^{IXa, AT-III}$ | 0.0000082 nM^-1^min^-1^ | (19) |
| $k_{a}^{Xa, AT-III}$ | 0.00015 nM^-1^min^-1^ | (20) |
| $k_{a}^{Xa, \alpha_{2}M}$ | 0.00004 nM^-1^min^-1^ | (21) |
| $k_{a}^{Xa, \alpha_{1}AT}$ | 0.0000136 nM^-1^min^-1^ | (21) |
| $k_{a}^{Xa, PCI}$ | 0.0012 nM^-1^min^-1^ | (22) |
| $k_{a}^{Xa-Va^{B}, AT-III}$ | 0.000022 nM^-1^min^-1^ | (21) |
| $k_{a}^{IIa, AT-III}$ | 0.00041 nM^-1^min^-1^ | (20) |
| $k_{a}^{IIa, \alpha_{2}M}$ | 0.0001 nM^-1^min^-1^ | (23) |
| $k_{a}^{IIa, \alpha_{1}AT}$ | 0.000003 nM^-1^min^-1^ | (24) |
| $k_{a}^{IIa, PCI}$ | 0.00037 nM^-1^min^-1^ | (22) |
| $k_{a}^{XIa, AT-III}$ | 0.000019 nM^-1^min^-1^ | (25) |
| $k_{a}^{XIa, \alpha_{2}AP}$ | 0.000026 nM^-1^min^-1^ | (25) |
| $k_{a}^{XIa, \alpha_{1}AT}$ | 0.000006 nM^-1^min^-1^ | (25) |
| $k_{a}^{XIa, PCI}$ | 0.0054 nM^-1^min^-1^ | (22) |
| $k_{a}^{XIa, C1I}$ | 0.00014 nM^-1^min^-1^ | (26) |
| $k_{a}^{APC, \alpha_{2}M}$ | 0.000006 nM^-1^min^-1^ | (27) |
| $k_{a}^{APC, \alpha_{2}AP}$ | 0.000006 nM^-1^min^-1^ | (27) |
| $k_{a}^{APC, \alpha_{1}AT}$ | 0.0000007 nM^-1^min^-1^ | (27) |
| $k_{a}^{APC, PCI}$ | 0.00039 nM^-1^min^-1^ | (22) |
| $h^{VIIIa}$ | 0.35 min^-1^ | (28) |
| $k_{eff}^{local Va, APC}$ | 7.7 nM^-1^min^-1^ | (29)* |
| $k_{eff}^{PC, APC}$ | 0.000282 nM^-1^min^-1^ | (30) |
| $K_{i}^{Va, PS}$ | 200 nM | (31)* |
| $K_{i}^{VIIIa, PS}$ | 150 nM | (32) |
| $k_{cat}^{PC, IIa}$, $K_{M}^{PC, IIa}$ | 1.2 min^-1^, 60000 nM | (33) |
|  | 5. Long-range feedback |  |
| $k_{eff}^{XI, IIa}$ | 0.03 nM^-2^min^-1^ | (34)* |
|  | 6. Platelet activation |  |
| $k_{cat}^{N, IIa}$,$K_{M}^{N, IIa}$ | 5.4 min^-1^, 2.4 nM | (35) |
| $n^{X(II)}$, $K_{d}^{X}$, $K_{d}^{II}$ | 16000 sites/platelet, 320 nM, 470 nM | (36) |
| $n^{VIIIa}$, $K_{d}^{VIIIa}$ | 750 sites/platelet, 1.5 nM | (37) |
| $n^{IXa}$, $K_{d}^{IXa}$ | 260 sites/platelet, 2.57 nM | (37) |
| $K_{d}^{Xa-Va}$ | 0.118 nM | (13) |
| $n^{Va}$, $K_{d}^{Va}$ | 2700 sites/platelet, 2.9 nM | (13;38) |
| $\rho^{V(a)}$ | 1000 molecules/platelet | (39)* |
|  | 7. Lysis |  |
| $k_{i}^{TPA, PAI1}$ | 1.5 nM^-1^min^-1^ | (40)* |
| $k_{a}^{TPA, Fn}$ | 6.9·10^-6^ nM^-1^min^-1^ | (41) |
| $k_{d}^{TPA_{B}}$ | 0.004 min^-1^ | (41) |
| $k_{cat}^{Pg\_G, Pn}$ | 3.4·10^-6^ nM^-1^min^-1^ | (42) |
| $k_{a}^{Pg\_G, Fn}$ | 6.5·10^-6^ nM^-1^min^-1^ | (43) |
| $k_{d}^{Pg\_G_{B}}$ | 0.25 min^-1^ | (43) |
| $k_{cat}^{Pg\_G, TPA}$ | 4·10^-5^nM^-1^min^-1^ | (44)* |
| $k_{a}^{Pg\_L, Fn}$ | 6.5·10^-6^ nM^-1^min^-1^ | (43) |
| $k_{d}^{Pg\_L_{B}}$ | 0.003 min^-1^ | (43) |
| $k_{cat}^{Pg\_L, TPA}$ | 5.4·10^-4^ nM^-1^min^-1^ | (44)* |
| $k_{i}^{Pg\_L, a2AP}$ | 0.06 nM^-1^min^-1^ | (45) |
| $k_{cat}^{Pg\_G_{B}, Pn}$ | 1.36·10^-4^nM^-1^min^-1^ | (42) |
| $k_{cat}^{Pg\_G_{B}, TPA_{B}}$ | 0.02 nM^-1^min^-1^ | (44)* |
| $k_{cat}^{Pg\_L_{B}, TPA_{B}}$ | 0.32 nM^-1^min^-1^ | (44)* |
| $k_{a}^{Pn, Fn}$ | 6·10^-6^nM^-1^min^-1^ | (46) |
| $k_{d}^{Pn_{B}}$ | 3·10^-3^ min^-1^ | (46) |
| $k_{i}^{Pn, a2AP}$ | 0.258 nM^-1^min^-1^ | (47) |
| $k_{i}^{Pn, a2M}$ | 0.018 nM^-1^min^-1^ | (41) |
| $k_{cat }^{Fn, Pn}$ | 300 min^-1^ | (48) |
| $\theta$ | 0.1^¶^ | - |
| $k_{i}^{Pn_{B}, a2AP}$ | 6·10^-4^ nM^-1^min^-1^ | (47) |

^*^ estimated value

^¶^ fitted value

## Table J. Model parameters: diffusion coefficients

| Model variable | *M*_r_ | Diffusion coefficient*, mm^2^/min |
| --- | --- | --- |
| VIIa | 50,000 | 0.0035 |
| VII. | 50,000 | 0.0035 |
| IXa | 46,000 | 0.0037 |
| IX | 57,000 | 0.0033 |
| Xa | 45,000 | 0.0037 |
| X | 58,500 | 0.0033 |
| IIa | 37,000 | 0.0040 |
| II | 72,000 | 0.0030 |
| Fn | — | 0 *^#^* |
| Fg | 340,000 | 0.0012 *^§^* |
| VIIIa | 160,000 | 0.0021 |
| VIII (+ vWF) | 240,000 (+ 500,000-20,000,000) | 0 ^¶^ |
| Va | 150,000 | 0.0022 |
| V | 330,000 | 0.0016 |
| APC | 62,000 | 0.0032 |
| PC | 62,000 | 0.0032 |
| Xa–TFPI | 95,000 | 0.0027 |
| TFPI | 40,000 | 0.0039 |
| AT-III | 58,000 | 0.0033 |
| XIa | 160,000 | 0.0021 |
| XI | 160,000 | 0.0021 |
| *N_a_* | ~10^13^ | 0|| |
| Pg_G | 92,000 | 0.0027 |
| Pg_L | 92,000 | 0.0027 |
| Pn | 85,000 | 0.0027 |
| TPA | 68,000 | 0.0029 |
| PAI1 | 52,000 | 0.0031 |
| a2M | 725,000 | 0.0006 |
| a2AP | 70,000 | 0.0029 |

* The values of diffusion coefficients were estimated on the basis of molecular weights of the components, using data from (49).

*^#^* Fibrin quickly polymerizes into fibrin net, and was therefore assumed not to diffuse.

*^§^* The value is from (49). Fibrinogen has non-globular shape, and therefore its estimation on the basis of molecular weight is incorrect.

^¶^ FVIII circulates in blood bound to von Willebrand factor, which exists in the form of huge complexes. We assumed these complexes not to diffuse.

^||^ Platelets were assumed not to diffuse.

Reference List

(1) Rodgers GM, Broze GJ, Jr., Shuman MA. The number of receptors for factor VII correlates with the ability of cultured cells to initiate coagulation. Blood 1984 Feb;63(2):434-8.

(2) Nemerson Y, Gentry R. An ordered addition, essential activation model of the tissue factor pathway of coagulation: evidence for a conformational cage. Biochemistry 1986 Jul 15;25(14):4020-33.

(3) Butenas S, Mann KG. Kinetics of human factor VII activation. Biochemistry 1996 Feb 13;35(6):1904-10.

(4) Rao LV, Williams T, Rapaport SI. Studies of the activation of factor VII bound to tissue factor. Blood 1996 May 1;87(9):3738-48.

(5) Komiyama Y, Pedersen AH, Kisiel W. Proteolytic activation of human factors IX and X by recombinant human factor VIIa: effects of calcium, phospholipids, and tissue factor. Biochemistry 1990 Oct 9;29(40):9418-25.

(6) Panteleev MA, Zarnitsina VI, Ataullakhanov FI. Tissue factor pathway inhibitor: a possible mechanism of action. Eur J Biochem 2002 Apr;269(8):2016-31.

(7) Gailani D, Ho D, Sun MF, Cheng Q, Walsh PN. Model for a factor IX activation complex on blood platelets: dimeric conformation of factor XIa is essential. Blood 2001 May 15;97(10):3117-22.

(8) Baugh RJ, Krishnaswamy S. Role of the activation peptide domain in human factor X activation by the extrinsic Xase complex. J Biol Chem 1996 Jul 5;271(27):16126-34.

(9) Krishnaswamy S, Field KA, Edgington TS, Morrissey JH, Mann KG. Role of the membrane surface in the activation of human coagulation factor X. J Biol Chem 1992 Dec 25;267(36):26110-20.

(10) Scandura JM, Walsh PN. Factor X bound to the surface of activated human platelets is preferentially activated by platelet-bound factor IXa. Biochemistry 1996 Jul 9;35(27):8903-13.

(11) Rawala-Sheikh R, Ahmad SS, Ashby B, Walsh PN. Kinetics of coagulation factor X activation by platelet-bound factor IXa. Biochemistry 1990 Mar 13;29(10):2606-11.

(12) Panteleev MA, Saenko EL, Ananyeva NM, Ataullakhanov FI. Kinetics of Factor X activation by the membrane-bound complex of Factor IXa and Factor VIIIa. Biochem J 2004 Aug 1;381(Pt 3):779-94.

(13) Tracy PB, Eide LL, Mann KG. Human prothrombinase complex assembly and function on isolated peripheral blood cell populations. J Biol Chem 1985 Feb 25;260(4):2119-24.

(14) van DG, Tans G, Rosing J, Hemker HC. The role of phospholipid and factor VIIIa in the activation of bovine factor X. J Biol Chem 1981 Apr 10;256(7):3433-42.

(15) Higgins DL, Lewis SD, Shafer JA. Steady state kinetic parameters for the thrombin-catalyzed conversion of human fibrinogen to fibrin. J Biol Chem 1983 Aug 10;258(15):9276-82.

(16) Hill-Eubanks DC, Lollar P. von Willebrand factor is a cofactor for thrombin-catalyzed cleavage of the factor VIII light chain. J Biol Chem 1990 Oct 15;265(29):17854-8.

(17) Monkovic DD, Tracy PB. Activation of human factor V by factor Xa and thrombin. Biochemistry 1990 Feb 6;29(5):1118-28.

(18) Baugh RJ, Broze GJ, Jr., Krishnaswamy S. Regulation of extrinsic pathway factor Xa formation by tissue factor pathway inhibitor. J Biol Chem 1998 Feb 20;273(8):4378-86.

(19) Pieters J, Willems G, Hemker HC, Lindhout T. Inhibition of factor IXa and factor Xa by antithrombin III/heparin during factor X activation. J Biol Chem 1988 Oct 25;263(30):15313-8.

(20) Rezaie AR. Calcium enhances heparin catalysis of the antithrombin-factor Xa reaction by a template mechanism. Evidence that calcium alleviates Gla domain antagonism of heparin binding to factor Xa. J Biol Chem 1998 Jul 3;273(27):16824-7.

(21) Ellis V, Scully M, MacGregor I, Kakkar V. Inhibition of human factor Xa by various plasma protease inhibitors. Biochim Biophys Acta 1982 Feb 4;701(1):24-31.

(22) Espana F, Berrettini M, Griffin JH. Purification and characterization of plasma protein C inhibitor. Thromb Res 1989 Aug 1;55(3):369-84.

(23) Jesty J. The kinetics of inhibition of alpha-thrombin in human plasma. J Biol Chem 1986 Aug 5;261(22):10313-8.

(24) Heeb MJ, Bischoff R, Courtney M, Griffin JH. Inhibition of activated protein C by recombinant alpha 1-antitrypsin variants with substitution of arginine or leucine for methionine358. J Biol Chem 1990 Feb 5;265(4):2365-9.

(25) Wuillemin WA, Eldering E, Citarella F, de Ruig CP, ten CH, Hack CE. Modulation of contact system proteases by glycosaminoglycans. Selective enhancement of the inhibition of factor XIa. J Biol Chem 1996 May 31;271(22):12913-8.

(26) Meijers JC, Vlooswijk RA, Bouma BN. Inhibition of human blood coagulation factor XIa by C-1 inhibitor. Biochemistry 1988 Feb 9;27(3):959-63.

(27) Heeb MJ, Gruber A, Griffin JH. Identification of divalent metal ion-dependent inhibition of activated protein C by alpha 2-macroglobulin and alpha 2-antiplasmin in blood and comparisons to inhibition of factor Xa, thrombin, and plasmin. J Biol Chem 1991 Sep 15;266(26):17606-12.

(28) Lollar P, Parker ET, Fay PJ. Coagulant properties of hybrid human/porcine factor VIII molecules. J Biol Chem 1992 Nov 25;267(33):23652-7.

(29) Solymoss S, Tucker MM, Tracy PB. Kinetics of inactivation of membrane-bound factor Va by activated protein C. Protein S modulates factor Xa protection. J Biol Chem 1988 Oct 15;263(29):14884-90.

(30) Hassouna H, Quinn C. Proteolysis of protein C in pooled normal plasma and purified protein C by activated protein C (APC). Biophys Chem 2002 Feb 19;95(2):109-24.

(31) Hackeng TM, van ', V, Meijers JC, Bouma BN. Human protein S inhibits prothrombinase complex activity on endothelial cells and platelets via direct interactions with factors Va and Xa. J Biol Chem 1994 Aug 19;269(33):21051-8.

(32) Koppelman SJ, Hackeng TM, Sixma JJ, Bouma BN. Inhibition of the intrinsic factor X activating complex by protein S: evidence for a specific binding of protein S to factor VIII. Blood 1995 Aug 1;86(3):1062-71.

(33) Esmon NL, DeBault LE, Esmon CT. Proteolytic formation and properties of gamma-carboxyglutamic acid-domainless protein C. J Biol Chem 1983 May 10;258(9):5548-53.

(34) Oliver JA, Monroe DM, Roberts HR, Hoffman M. Thrombin activates factor XI on activated platelets in the absence of factor XII. Arterioscler Thromb Vasc Biol 1999 Jan;19(1):170-7.

(35) Smith RD, Owen WG. Platelet responses to compound interactions with thrombin. Biochemistry 1999 Jul 13;38(28):8936-47.

(36) Scandura JM, Ahmad SS, Walsh PN. A binding site expressed on the surface of activated human platelets is shared by factor X and prothrombin. Biochemistry 1996 Jul 9;35(27):8890-902.

(37) Ahmad SS, Scandura JM, Walsh PN. Structural and functional characterization of platelet receptor-mediated factor VIII binding. J Biol Chem 2000 Apr 28;275(17):13071-81.

(38) Tracy PB, Nesheim ME, Mann KG. Platelet factor Xa receptor. Methods Enzymol 1992;215:329-60.

(39) Baruch D, Hemker HC, Lindhout T. Kinetics of thrombin-induced release and activation of platelet factor V. Eur J Biochem 1986 Jan 2;154(1):213-8.

(40) Chmielewska J, Ranby M, Wiman B. Kinetics of the inhibition of plasminogen activators by the plasminogen-activator inhibitor. Evidence for 'second-site' interactions. Biochem J 1988 Apr 15;251(2):327-32.

(41) Higgins DL, Vehar GA. Interaction of one-chain and two-chain tissue plasminogen activator with intact and plasmin-degraded fibrin. Biochemistry 1987 Dec 1;26(24):7786-91.

(42) Suenson E, Thorsen S. The course and prerequisites of Lys-plasminogen formation during fibrinolysis. Biochemistry 1988 Apr 5;27(7):2435-43.

(43) Lucas MA, Fretto LJ, McKee PA. The binding of human plasminogen to fibrin and fibrinogen. J Biol Chem 1983 Apr 10;258(7):4249-56.

(44) Ranby M. Studies on the kinetics of plasminogen activation by tissue plasminogen activator. Biochim Biophys Acta 1982 Jun 24;704(3):461-9.

(45) Wiman B, Lijnen HR, Collen D. On the specific interaction between the lysine-binding sites in plasmin and complementary sites in alpha2-antiplasmin and in fibrinogen. Biochim Biophys Acta 1979 Jul 25;579(1):142-54.

(46) Suenson E, Thorsen S. Secondary-site binding of Glu-plasmin, Lys-plasmin and miniplasmin to fibrin. Biochem J 1981 Sep 1;197(3):619-28.

(47) Kolev K, Lerant I, Tenekejiev K, Machovich R. Regulation of fibrinolytic activity of neutrophil leukocyte elastase, plasmin, and miniplasmin by plasma protease inhibitors. J Biol Chem 1994 Jun 24;269(25):17030-4.

(48) Wu JH, Diamond SL. A fluorescence quench and dequench assay of fibrinogen polymerization, fibrinogenolysis, or fibrinolysis. Anal Biochem 1995 Jan 1;224(1):83-91.

(49) Marshal AG. Biological chemistry: principles, technics, and applications . New York: John Wiley and Sons ; 1978.
